# A Computational Model of the Rainbow Trout Hypothalamus-Pituitary-Ovary-Liver Axis

**DOI:** 10.1371/journal.pcbi.1004874

**Published:** 2016-04-20

**Authors:** Kendall Gillies, Stephen M. Krone, James J. Nagler, Irvin R. Schultz

**Affiliations:** 1 Battelle, Pacific Northwest National Laboratory, Marine Sciences Laboratory, Sequim, Washington, United States of America; 2 University of Idaho, Department of Mathematics, Moscow, Idaho, United States of America; 3 University of Idaho, Department of Biological Sciences and Center for Reproductive Biology, Moscow, Idaho, United States of America; Johns Hopkins University, UNITED STATES

## Abstract

Reproduction in fishes and other vertebrates represents the timely coordination of many endocrine factors that culminate in the production of mature, viable gametes. In recent years there has been rapid growth in understanding fish reproductive biology, which has been motivated in part by recognition of the potential effects that climate change, habitat destruction and contaminant exposure can have on natural and cultured fish populations. New approaches to understanding the impacts of these stressors are being developed that require a systems biology approach with more biologically accurate and detailed mathematical models. We have developed a multi-scale mathematical model of the female rainbow trout hypothalamus-pituitary-ovary-liver axis to use as a tool to help understand the functioning of the system and for extrapolation of laboratory findings of stressor impacts on specific components of the axis. The model describes the essential endocrine components of the female rainbow trout reproductive axis. The model also describes the stage specific growth of maturing oocytes within the ovary and permits the presence of sub-populations of oocytes at different stages of development. Model formulation and parametrization was largely based on previously published in vivo and in vitro data in rainbow trout and new data on the synthesis of gonadotropins in the pituitary. Model predictions were validated against several previously published data sets for annual changes in gonadotropins and estradiol in rainbow trout. Estimates of select model parameters can be obtained from in vitro assays using either quantitative (direct estimation of rate constants) or qualitative (relative change from control values) approaches. This is an important aspect of mathematical models as in vitro, cell-based assays are expected to provide the bulk of experimental data for future risk assessments and will require quantitative physiological models to extrapolate across biological scales.

## Author Summary

Reproduction in fishes and other vertebrates represents the timely coordination of many endocrine factors that culminate in the production of mature, viable gametes. Improving the ability to estimate reproductive performance in fish is important, due to the growth of the aquaculture industry and the need to maintain adequate broodstock and concerns over the effects of anthropogenic stressors on feral fish populations. We present here a quantitative, mathematical model of the female rainbow trout reproductive cycle. We show how the model is able to accurately describe experimentally measured data associated with pituitary, ovarian and liver reproductive performance. We also use the model to describe similar data sets collected in rainbow trout by other researchers. An important value of quantitative biological models is the ability to simulate various physiological conditions, real or hypothetical. We demonstrate this by predicting the effects of exposure to an endocrine disruptor on oocyte growth. The need to limit cost and animal usage will encourage future experimental studies to use in vitro methods. The model presented here can assist with the extrapolation of in vitro effects to the whole fish.

## Introduction

**Fig 1:**
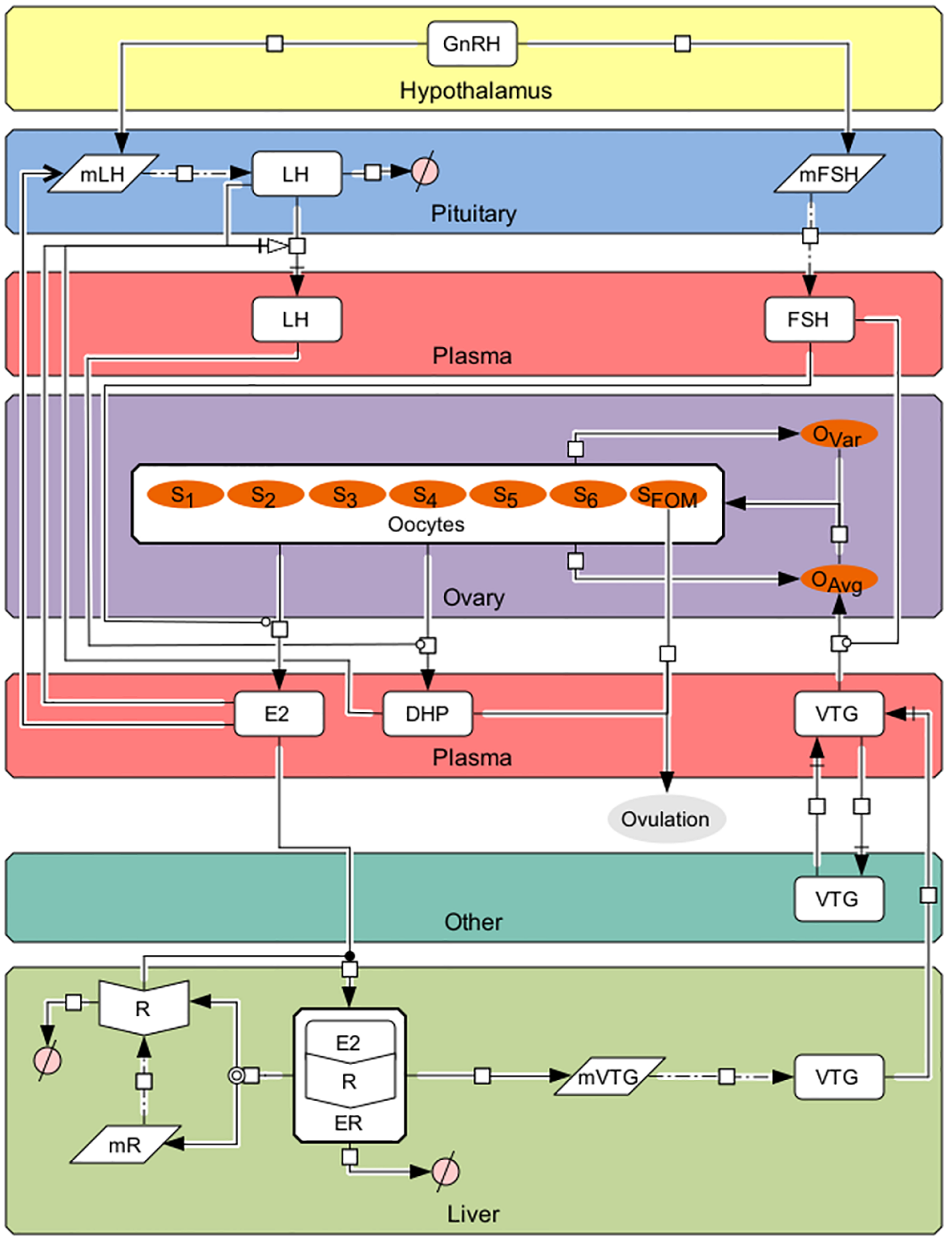
The HPOL signaling network in rainbow trout as formulated in our model. Arrows and symbols on graph follow CellDesigner vs. 4.4 notation (www.celldesigner.org). GnRH is secreted from the hypothalamus into the pituitary stimulating the production of mFSH and mLH, which then leads to formation of FSH and LH, respectively. FSH, which is being continuously secreted from the pituitary, travels to the ovaries to stimulate production of E2. E2 then travels to the liver to bind with E2 receptors (R; translated from mR) to form ER. ER then stimulates the production of mVTG, which produces VTG_L_. Secreted VTG then travels from the liver to the ovaries via the plasma (VTG_P_) where it is absorbed by follicles in stages 3 through 6 (the proportion of follicles in these stages are denoted by S_j_, j = 3, 4, 5, and 6) during vitellogenesis, the rate of which is affected by FSH_P_, to promote oocyte growth (O_Avg_). Oocyte growth then progresses the oocytes through the stages using a Weibull distribution created from O_Avg_ together with O_Var_. In the later stages LH_P_ stimulates the oocytes to produce DHP. Finally, oocytes undergo final maturation (S_FOM_) and combined with DHP, determine when the fish ovulates. Description of symbols are found in Table 1.

Fishes, like other vertebrates, have an endocrine signaling network operating between the brain and the gonads controlling reproduction. This network spans the hypothalamus, the pituitary, the gonads and, in fishes and other egg-laying vertebrates, the liver, and is referred to in this paper as the hypothalamus-pituitary-ovary-liver (HPOL) axis (Fig 1 and Table 1) [1]. Communication between these components takes place via the blood as well as through neurosecretory fibers from the hypothalamus to the pituitary [2]. The HPOL axis begins in the hypothalamus where environmental cues trigger the production of gonadotropin-releasing hormone (GnRH) [3]. GnRH is then sent to the pituitary where it stimulates the synthesis of two gonadotropins (GTHs): follicle-stimulating hormone (FSH) and luteinizing hormone (LH) [4]. Both GTHs regulate production of sex steroids by ovarian follicles, where the follicles are defined as the oocyte and the surrounding cell layers [5,6]. FSH stimulates the somatic cell layers of the follicle to produce estradiol-17β (E2) [7], which stimulate the liver to produce the egg-yolk protein vitellogenin (VTG). VTG is subsequently secreted into the bloodstream and incorporated into the developing oocyte, the accumulation of which is the primary cause for oocyte growth. LH is involved in the later stages of oocyte development and stimulates the somatic cell layers of the follicles to produce a maturation-inducing steroid, which in rainbow trout (*Onchorynchus mykiss*) and many other fishes is 17α, 20β-dihydroxy-4-pregnen-3-one (DHP). DHP is the primary hormone involved in the final stages of oocyte maturation and ovulation [8]. Beyond these functions, the above five hormones interact with each other through positive and negative feedback loops, creating an intricate network of pathways that leads to the synchronization of processes culminating in the timely production of mature eggs.

**Table 1:** Variable names.

Variable	Definition	Units
GnRH	Gonadotropin releasing hormone	unitless
mFSH	FSH_β_ subunit mRNA	pg/μg of RNA
mLH	LH_β_ subunit mRNA	pg/μg of RNA
LH_Pit_	Pituitary levels of luteinizing hormone	ng/mg of pituitary
LH_P_	Plasma levels of luteinizing hormone	ng/ml
FSH_P_	Plasma levels of follicle stimulating hormone	ng/ml
S_J_	Proportion of follicles in stage j (j = 1,2,…,6,FOM)	unitless
O_AVG_	Average follicle diameter	mm/kg
O_VAR_	Variance of follicle diameters	mm^2^
E2	Estradiol-17β	ng/ml
DHP	17α, 20β-dihydroxy-4-pregnen-3-one	ng/ml
mR	Estrogen receptor mRNA	pg/μg of RNA
R	Estrogen receptor	fmol/g of liver
ER	Estrogen receptor complex	fmol/g of liver
mVTG	Vitellogenin mRNA	pg/μg of RNA
VTG_L_	Liver levels of vitellogenin	mg/g of liver
VTG_N_	Level of vitellogenin in compartments other than the plasma, liver, and ovaries	mg/ml
VTG_P_	Plasma levels of vitellogenin	mg/ml

The overall complexity of the fish reproductive axis is on a level similar to mammalian systems; however, development of mathematical descriptions for fish has only occurred relatively recently. One challenge with modeling the fish reproductive axis is that spawning behavior generally occurs in two forms: group synchronous spawning (a single large clutch of oocytes develop synchronously for one spawning event; e.g. salmon, trout) or asynchronous spawning (several small clutches of eggs are spawned at different times during a reproductive season; e.g. zebrafish [*Danio rerio*], fathead minnow [*Pimephales promelas*], medaka [*Oryzias latipes*]). Although the same set of reproductive hormones appear to be involved in both types of fishes, the roles of some hormones, such as the GTHs, are not as well defined in asynchronous compared to synchronous spawning fishes. This and other factors associated with oocyte development have made conceptualization of models distinct between the two spawning types. In group synchronous spawning fishes an early pituitary-gonad-liver model for the Atlantic croaker (*Micropogonias undulates*) was described [9,10] and included descriptions of production for a single GTH (combined FSH\LH), E2 formation and subsequent VTG production. The model was then used to predict the effects of chemical exposures (a PCB mixture and cadmium) and environmental stress (prolonged hypoxia) on the pituitary-gonad-liver axis [9,10]. A model for the coho salmon (*O*. *kisutch*) hypothalamus-pituitary-gonad axis was described [11], which added a simple description of oocyte maturation, synthesis of DHP and separate descriptions for FSH and LH. A more detailed description of vitellogenesis throughout the reproductive cycle was described for rainbow trout [12] and included parameters for the expression of mRNA coding for the estrogen receptor and VTG. A specialized model for the unnatural stimulation of vitellogenesis in male trout was presented [13], which focused on transcriptional imprinting of VTG expression. In asynchronous spawning fish, greatest attention has been placed on the fathead minnow and a focus towards its importance as an ecotoxicological test species. A series of pituitary-gonad-liver models for this species have been described [14,15,16,17]. These models included empirical descriptions of a single GTH variable and subsequent stimulation of testosterone and E2 synthesis in the gonads. Model formulation was integrated with toxicokinetic descriptions of E2 or ethynylestradiol (EE2; synthetic E2 agonist) exposure to male minnows [14], EE2 and 17β-trenbolone (synthetic testosterone agonist) to female minnows [16] or fadrozole (E2 synthesis inhibitor) to female minnows [15,17]. These models were used to characterize the reproductive effects of exposure to sex steroid agonists or antagonists to fathead minnows. Other more specialized models for the fathead minnow include the model by [18] for E2 synthesis, which characterizes the multiple steps involved in the conversion of cholesterol to E2, and the model by [19], which models the process of asynchronous oocyte development by tracking multiple clutches of oocytes through a maturation cycle and spawning. The emphasis in modeling E2 and VTG production in fathead minnows reflects the critical role these variables play in controlling fecundity and successful spawning [20].

The growing interest in modeling the fish reproductive axis is due in part to awareness that accelerating climate change, habitat destruction and discharging of contaminants into waterways have potential effects on natural and cultured fish populations. New approaches to understanding the impacts of these stressors are being developed that require more biologically accurate and detailed mathematical models [21,22]. The value of these models for mammalian systems has been well-demonstrated and resides in their potential to aid in understanding biological control processes associated with reproduction and test hypotheses that may be difficult or impractical to do in vivo [23]. The objective of the present study is to present a comprehensive model for the HPOL axis for female rainbow trout that can be used to assess impacts of physical and chemical stressors on reproduction. We combine elements of past models [11,12] with new features that better characterize processes associated with oocyte growth and maturation.

## Methods

### Ethics statement

All experiments were performed according to the guidelines established by the Institutional Animal Care and Use Committee (IACUC) of PNNL.

### Background

The central focus of the model is on the progression of events that lead to oocyte growth through several stages, followed by final oocyte maturation (FOM) and then ovulation, whose timing is taken here to be the time point at which DHP first exceeds a threshold and when almost all of the oocytes are in FOM. We do not include actual spawning in the model because the release of eggs is different in the wild (where it is triggered by interactions with male fish) and in aquaculture (where the eggs are usually “stripped” by hand). In the model, concentrations of FSH, LH and VTG are given for separate tissue compartments. To differentiate between compartments subscripts are used: blood plasma is denoted with a P, the pituitary is denoted with a Pit, the ovary with a O and the liver is denoted with an L. Other tissues not specified are denoted with an N. The description of the equations for a hormone’s plasma levels occurs in the compartment associated with its synthesis and secretion. A clearance–volume approach is used to describe the pharmacokinetic behavior of VTG and circulating hormones, with a plasma referenced volume of distribution and a clearance parameter that represents loss due to excretion or sequestration (e.g. VTG accumulation in oocytes). The volume of distribution of a hormone is denoted by V_i_ and plasma clearance of a hormone is denoted by Cl_i_, where i is replaced with the hormone name, e.g. FSH, LH, E2. In addition to the previously mentioned parameters, a consistent notation is used for parameters performing the same biological function, e.g. synthesis and degradation, to help maintain model uniformity.

### Functions

The model uses Hill and Heaviside (a.k.a. the Unit Step function) functions to describe nonlinear hormone interactions. The Hill function is a sigmoidal function with values ranging from 0 to 1 and is used to model stimulatory (H_+_) and inhibitory (H_-_) effects that have a threshold,

$$H_{+}(x,T,n)=\frac{x^{n}}{x^{n}+T^{n}}\text{ and }H_{-}(x,T,n)=\frac{T^{n}}{x^{n}+T^{n}}.$$ (1)

In each equation x is the concentration of the hormone, protein, or mRNA, T is the threshold value at which x exerts half of its maximal feedback and n determines the range of concentrations x must fall in to switch between no effect and maximal effect. Values of x not close to T will fall outside of the interval T±ε_n_, where ε_n_ is a positive constant depending on n and is a mathematical representation of the maximal distance a value can be from T and still be considered close. For values of x outside this interval the Hill function remains relatively close to zero, no effect, or relatively close to one, maximal effect. As n becomes larger ε_n_ becomes smaller and the change between no effect and the maximal effect becomes more sudden; letting n go to infinity results in an instantaneous switch between no effect, zero, and the maximal effect, one, when concentration levels of x reach T. This on/off switch can be modeled using the Heaviside function,

$$U(t)=\{\begin{array}{ll} 0 & t\leq 0\\ 1 & t>0 \end{array},$$ (2)

by evaluating it at x-T when letting n go to infinity in H_+_ or T-x when letting n go to infinity in H_-_.

To incorporate time delays in interactions between model variables, transit compartments are used (reviewed in [24]). Transit compartments have been shown to be well suited to account for delays associated with signal transduction processes [25], which for example, occurs in the present model with FSH stimulation of E2 synthesis. This approach allows for a less complex model but still retains the capacity to incorporate more details of the signal transduction process, which can directly replace transit compartments. An individual transit compartment is restricted to short time delays, so for longer time delays that indicate multiple distinct processes are occurring, more than one transit compartment is used. Furthermore, the number of distinct processes can increase or decrease based on level of detail describing the biological process. This allows us to not only find the optimal number of transit compartments to use to represent the delay of hormone A’s influence on hormone B by D_A,B_ hours, denoted by m, but it is also reasonable to assume the delay produced by an individual transit compartment is equal to the average delay of the compartments, D_A,B_/m. A superscript of TC_m_ is used for a system of m transit compartments; for example, if the growth of B starting at time 0 is modeled by

$$\begin{array}{ll} \frac{d}{dt}\left[B\right]=c_{1}\left[A\right]-c_{2}\left[B\right]\text{,} & \left[B\right]\left(0\right)=B_{0} \end{array}$$ (3)

then incorporating the time delay using a system of m transit compartments will change the formula to

$$\begin{array}{ll} \frac{d}{dt}\left[B\right]=c_{1}\left[A^{TC_{m}}\right]-c_{2}\left[B\right]\text{,} & \left[B\right]\left(0\right)=B_{0} \end{array}$$ (4)

where the transit compartments are given by

$$\begin{array}{cccc} \frac{d}{dt}\left[A^{TC_{1}}\right] & = & \frac{m}{D_{A,B}}\left(\left[A\right]-\left[A^{TC_{1}}\right]\right), & \left[A^{TC_{1}}\right]\left(0\right)=\left[A\right]\left(t_{0}\right)\\ \frac{d}{dt}\left[A^{TC_{2}}\right] & = & \frac{m}{D_{A,B}}\left(\left[A^{TC_{1}}\right]-\left[A^{TC_{2}}\right]\right), & \left[A^{TC_{2}}\right]\left(0\right)=\left[A\right]\left(t_{0}\right)\\ & \vdots & & \\ \frac{d}{dt}\left[A^{TC_{m}}\right] & = & \frac{m}{D_{A,B}}\left(\left[A^{TC_{m-1}}\right]-\left[A^{TC_{m}}\right]\right), & \left[A^{TC_{m}}\right]\left(0\right)=\left[A\right]\left(t_{0}\right) \end{array}.$$ (5)

The use of transit compartments, Hill functions and the Heaviside function help describe delays and nonlinear interactions between hormones and key reproductive processes.

### Hypothalamus—Pituitary

The hypothalamic region of the mid-brain is well established as the signal initiation site that stimulates the pituitary gland at the base of the brain to release GTHs that act on the gonads to cause their sexual development. The endocrine control of the HPOL pathway begins in neurosecretory fibers from the hypothalamus that project directly into the adenohypophysis region of the pituitary gland [26]. These fibers release GnRH in the vicinity of the gonadotrophs, the pituitary cells that produce the GTHs, increasing the basal synthesis rate of the beta subunit mRNA for FSH (mFSH) and LH (mLH). The GTHs are heterodimeric proteins consisting of a common α and GTH specific β subunit.

The regulation of GnRH is partly due to kisspeptins (KISS), which are the links from environmental cues to the HPOL axis [2]. The complexity of the GnRH-KISS system is still being characterized in fish and therefore we have chosen to not include it in this version of the model. We have assumed that FSH beta subunit mRNA levels primarily reflect changes in GnRH released into the pituitary during the reproductive cycle. Thus, an empirical equation for GnRH production was derived based on previously measured mFSH levels in the trout pituitary. The equations for mFSH and mLH are based on the equations found in [11] and begin with a basal synthesis rate, k_s,mFSH_ and k_s,mLH_, and a degradation rate, k_d,mFSH_ and k_d,mLH_. The positive feedback of GnRH is represented through a stimulatory factor, α_mFSH,GnRH_ and α_mLH,GnRH_. E2 is known to increase the expression of mLH and pituitary content of LH [27,28,29]. This positive feedback effect of E2 on mLH is represented though a stimulatory factor, α_mLH,E2_, and delayed through the use of transit compartments by D_E2,mLH_ hours to account for signal transduction and other biological processes.

$$\frac{d}{dt}\left[mFSH\right]=k_{s,mFSH}\left(1+\alpha_{mFSH,GnRH}\cdot GnRH(t)\right)-k_{d,mFSH}\left[mFSH\right]$$ (6)

$$\frac{d}{dt}\left[mLH\right]=k_{s,mLH}\left(1+\alpha_{mLH,GnRH}\cdot GnRH(t)+\alpha_{mLH,E2}\left[E2^{TC_{m}}\right]\right)-k_{d,mLH}\left[mLH\right]$$ (7)

After FSH is synthesized from mFSH it is immediately released into the blood. The tight coupling of mFSH levels and blood FSH levels ([30], and data presented in this study) allows the model to have a constant rate of synthesis and release of FSH from the pituitary, k_s,FSH_, adjusted by the weight of the pituitary, w_Pit_. As in [11], the clearance-volume approach then leads to the following equation:

$$\frac{d}{dt}\left[FSH_{P}\right]=\frac{1}{V_{FSH}}\left(w_{Pit}\cdot k_{s,FSH}\left[mFSH\right]-Cl_{FSH}\left[FSH_{P}\right]\right).$$ (8)

Plasma levels of FSH are detectible throughout the reproductive cycle, implying a continuous release from the pituitary; in contrast, plasma levels of LH remain largely undetectable until the end of the reproductive cycle. Though plasma levels of LH remain undetectable, pituitary levels continue to increase [30] implying a block on the release of LH from the pituitary into the plasma. The release of LH from the pituitary is known to be controlled by a dopamine (D2) inhibition, which is strengthened by E2 through positive feedback on D2 receptors (D2r) in the pituitary [31,32]. Declining levels of E2 remove the block on LH release [32] only after levels surpass a threshold, T_E2,LH_, [33]. Besides E2, rising DHP levels also appear to block LH release. In some fishes, DHP causes an increase of D2 receptors in the pituitary [31], and in rainbow trout, DHP significantly decreased the release of LH in-vitro [34]. To model the release of LH from the pituitary we have chosen a release function that assumes the following: for each ng/ml of LH in the pituitary there exists a minimum amount of D2 receptors needed to maintain the block, the amount of LH released depends on the pituitary levels of LH that do not have the necessary amount of D2r to be blocked, the quantity of D2 receptors is proportional to levels of E2 and DHP and for the block to be removed, E2 levels must be higher than a threshold T_E2,LH_. To better understand how these assumptions lead to the final release function found in Eq (12) an initial release function will be created from the first assumption, Eq (9), and built upon from the remaining assumptions, Eq (10) through Eq (12). The first assumption can be interpreted as the release of LH occurs when the amount of LH in the pituitary exceeds the minimum amount of D2-receptors per ng/ml of LH in the pituitary, ξ where ξ>0, e.g.

$$R_{LH}(t)=\{\begin{array}{cc} 0 & \left[LH_{P}\right]\leq \left[D2r\right]/\xi \\ 1 & \left[LH_{P}\right]>\left[D2r\right]/\xi \end{array}=U\left(\left[LH_{Pit}\right]-\left[D2r\right]/\xi \right).$$ (9)

Applying the second and third assumption to the release function gives

$$R_{LH}(t)=\Theta (t)\cdot U\left(\Theta (t)\right),$$ (10)

where

$$\Theta (t)=\left[LH_{Pit}\right]-N_{E2}\left[E2\right]-N_{DHP}\left[DHP\right]$$ (11)

and [D2r]/ ξ = N_E2_[E2]+N_DHP_[DHP]. Finally, the fourth assumption gives the final release function of

$$R_{LH}(t)=\Theta (t)\cdot U\left(\Theta (t)\right)\cdot H_{+}\left(\left[E2\right],T_{E2,LH},n_{E2,LH}\right).$$ (12)

With the exception of the release function the equations modeling the pituitary and plasma levels of LH are similar to those found in [11]. In the pituitary LH is synthesized from mLH at a constant rate of k_s,LH_. The stored LH is then subject to degradation at a rate of k_d,LH_ before it is released at a rate of k_r,LH_. The LH released into the plasma is adjusted by the weight of the pituitary, w_Pit_, and follows the clearance-volume approach.

$$\frac{d}{dt}\left[LH_{Pit}\right]=k_{s,LH}\left[mLH\right]-k_{d,LH}\left[LH_{Pit}\right]-k_{r,LH}\cdot R_{LH}(t)$$ (13)

$$\frac{d}{dt}\left[LH_{P}\right]=\frac{1}{V_{LH}}\left(w_{Pit}\cdot k_{r,LH}\cdot R_{LH}(t)-Cl_{LH}\left[LH_{P}\right]\right)$$ (14)

After release into the plasma, the GTHs travel to the ovaries to stimulate production of the sex steroids.

### Ovary

The process of oocyte growth can be initially separated according to the capacity to absorb vitellogenin: pre-vitellogenic, vitellogenic and post-vitellogenic. These stages can be further subdivided according to specific morphological and biochemical changes occurring during growth [6]. Our model divides the reproductive cycle into seven stages; stages 1 and 2 are pre-vitellogenic, stages 3 through 6 are vitellogenic and the last stage is FOM. The proportion of oocytes in stage j, j = 1,2,…,6,FOM, at time t will be denoted by S_j_(t). The model in [11] characterizes the stage of an oocyte based on length of time in the reproductive process and did not contain feedback from other variables. In the present model the stage of an oocyte is based on its size, which is defined by its diameter, and interacts with other variables. This is a convenient metric that is commonly reported in experimental studies and has been used to determine oocyte staging in trout (see Appendix S1 for ways to translate diameter to other measurements of size). The distribution of diameters at time t is given by a Weibull distribution with time dependent parameters l(t) and k(t). The distribution's parameters are chosen such that the mean and variance of the Weibull distribution at time t is equal to the mean and variance of oocyte sizes given by Eqs (16) and (18), respectively. Hence the stages are given by

$$S_{j}(t)=\{\begin{array}{ll} P(0\leq X_{t}\leq s_{j}) & j=1\\ P(s_{j-1}\leq X_{t}\leq s_{j}) & j=2,\ldots ,6\\ P(s_{6}\leq X_{t}) & j=FOM \end{array}$$ (15)

where s_j_ is the boundary size between stages j-1 and j and the random variable X_t_ is the size of an oocyte at time t with *X*_*t*_ ~ *Weibull*(*l*(*t*),*k*(*t*)).

When describing oocyte growth it is important to recognize that approximately 84% of the increase in oocyte diameter occurs during vitellogenesis [5]. Therefore, the model assumes that oocyte growth rate is dependent on the amount of VTG being sequestered, Seq(t), and the conversion of diameter increase for every unit of VTG sequestered,$k_{V,O_{Avg}}$. The growth rate in the non-vitellogenic stages is described by a constant growth rate,$k_{NV,O_{Avg}}$, as only a small portion of growth occurs during these stages.

$$\frac{d}{dt}\left[O_{Avg}\right]=k_{NV,O_{Avg}}\cdot \left(S_{1}(t)+S_{2}(t)+S_{FOM}(t)\right)+k_{V,O_{Avg}}\cdot Seq(t)\left[VTG_{P}\right]$$ (16)

The rate at which the oocytes absorb VTG is determined in part by circulating levels of FSH, oocyte stage, and VTG levels [35,36,37]. The increase in basal sequester rate, Cl_VTG,seq_, due to circulating FSH is represented with a Hill function with a half threshold of T_Seq,FSH_. Since oocytes can only sequester VTG when they exceed a certain size, [37], and have not entered FOM, the sequester period will only be from stage 3 until stage 6.

$$Seq(t)=Cl_{VTG,Seq}\left(1+H_{+}\left(\left[FSH_{P}\right],T_{Seq,FSH},1\right)\right)\sum_{j=3}^{6}S_{j}(t).$$ (17)

The growth rate of individual oocytes can vary substantially, presumably due to differential uptake of VTG but other, unknown factors may also be involved. At the start of the reproductive cycle, all primary oocytes (stage 1) appear to be uniform in size. As oocytes mature towards the mid-vitellogenic stage, size variance increases before becoming more uniform as final maturation nears [38]. To model this, each stage will have a maximal variance assigned to it,$\alpha_{O_{Var},S_{j}}$, where the variances increase as the oocytes near the mid-vitellogenic stage, Stage 4, and decrease as the oocytes approach FOM.

$$O_{Var}\left(t\right)=\sum_{j\in K}\alpha_{O_{Var},S_{j}}S_{j}(t)\text{ where }K=\left\{1,2,\ldots ,6,FOM\right\}$$ (18)

During stages 2 through FOM oocytes produce E2 and DHP when FSH and LH, respectively, are introduced to the ovaries. Once in the ovaries FSH and LH stimulate the thecal and granulosa cells, the layers of somatic cells surrounding the oocytes, to produce E2 and DHP, respectively [1]. When FSH or LH binds to their respective receptors, the ensuing stimulation of steroid synthesis is not instantaneous. To account for this delay, transit compartments are introduced in place of FSH and LH in Eqs (19) and (20), respectively, with time delays D_FSH,E2_ and D_LH,DHP_, respectively. The sensitivity of the oocytes' responses to LH and FSH is stage specific and the amount of steroid secreted into the plasma can be thought of as a clearance from the follicle. Hence a follicle in stage j will produce $Cl_{E2,S_{j}}$ ($Cl_{DHP,S_{j}}$) ml of E2 (DHP) for each ng/ml of FSH (LH) that stimulates. Therefore, the total amount of E2 produced during Stage j at time t is $Cl_{E2,S_{j}}\cdot n_{oocyte}\cdot S_{j}(t)\cdot \left[FSH_{P}^{TC_{n}}\right]$, where n_oocyte_ is the average number of oocytes per kg of fish. Similarly, the total amount of DHP produced during Stage j at time t is $Cl_{DHP,S_{j}}\cdot n_{oocyte}\cdot S_{j}(t)\cdot \left[LH_{P}^{TC_{n}}\right]$. In addition to FSH stimulated E2 production, we have a basal production rate for each follicle, k_E2_. We do not include a basal production rate for DHP since the follicles ability to produce DHP is only a short time period.

$$\frac{d}{dt}\left[E2\right]=\frac{1}{V_{E2}}\left\{n_{oocyte}\left(k_{E2}+\left[FSH_{P}^{TC_{m}}\right]\sum_{j=2}^{6}Cl_{E2,S_{j}}S_{j}(t)\right)-Cl_{E2}\left[E2\right]\right\}$$ (19)

$$\frac{d}{dt}\left[DHP\right]=\frac{1}{V_{DHP}}\left(n_{oocyte}\cdot \left[LH_{P}^{TC_{m}}\right]\sum_{j\in L}Cl_{DHP,S_{j}}S_{j}(t)-Cl_{DHP}\left[DHP\right]\right)\text{ where }L=2,\ldots ,6,FOM$$ (20)

Once produced, E2 and DHP are then released into the blood and stimulate the production of VTG and promote ovulation, respectively.

### Liver

The liver plays an essential role in the reproductive process because it synthesizes the egg yolk precursor protein VTG, which is necessary for oocyte growth and development [39]. Our model for VTG synthesis is largely based on the model described by [12]. VTG synthesis is initiated when E2 binds to a nuclear estrogen receptor, R, at a binding rate of k_on,ER_, which is produced by transcription of the estrogen receptor mRNA, mR. The resulting E2-receptor complex, ER, stimulates the production of mR in a positive auto-regulatory feedback loop and stimulates the production of the mRNA for VTG, mVTG. Based on the experimental data of [40] we assume that activation of transcription happens on a one-to-one ratio, meaning a given dose of E2 yields similar increases in the E2-receptor complex, ER, and mVTG levels. ER then dissociates at a rate of k_off,ER_ increasing the number of unbound E2-receptors, R.

$$\frac{d}{dt}\left[mR\right]=k_{s,mR}\left(1+\alpha_{mR,ER}\left[ER\right]\right)-k_{d,mR}\left[mR\right]$$ (21)

$$\frac{d}{dt}\left[R\right]=k_{s,R}\left[mR\right]-k_{d,R}\left[R\right]-k_{on,ER}\left[E2\right]\cdot \left[R\right]+k_{off,ER}\left[ER\right]$$ (22)

$$\frac{d}{dt}\left[ER\right]=k_{on,ER}\left[E2\right]\cdot \left[R\right]-\left(k_{off,ER}+k_{d,ER}\right)\left[ER\right]$$ (23)

$$\frac{d}{dt}\left[mVTG\right]=k_{s,mVTG}\left(1+\alpha_{mVTG,ER}\left[ER\right]\right)-k_{d,mVTG}\left[mVTG\right]$$ (24)

Once synthesized, each molecule of mVTG can be translated into VTG multiple times; this ability is represented by the amplification factor γ. Furthermore, N_mVTG_ represents a scaling factor for mVTG concentration. The VTG in the liver, denoted by VTG_L_, is secreted into the plasma at a rate of k_r,VTG_, the amount of which is denoted by VTG_P_. The amount of VTG released into the plasma is measured in g/kg of liver weight, w_L_. VTG is then exchanged between the plasma and compartments other than the liver and ovaries, VTG_N_, taken up by the ovaries through the oocytes, Eq (17), or cleared from the body, Cl_VTG_. The VTG in compartments other than the ovary are assumed to be in reversible equilibrium with blood with a transfer clearance of Cl_VTG,trans_.

$$\frac{d}{dt}\left[VTG_{L}\right]=k_{s,VTG}{\left(\frac{\left[mVTG\right]}{N_{mVTG}}\right)}^{\gamma }-k_{r,VTG}\left[VTG_{L}\right]$$ (25)

$$\frac{d}{dt}\left[VTG_{N}\right]=\frac{1}{V_{VTG_{N}}}\left(Cl_{VTG,trans}\left[VTG_{P}\right]-Cl_{VTG,trans}\left[VTG_{N}\right]\right)$$ (26)

$$\frac{d}{dt}\left[VTG_{P}\right]=\frac{1}{V_{VTG_{P}}}\left(k_{r,VTG}\cdot w_{L}\left[VTG_{L}\right]+Cl_{VTG,trans}\left[VTG_{N}\right]-\left(Cl_{VTG,trans}+Seq(t)+Cl_{VTG}\right)\left[VTG_{P}\right]\right)$$ (27)

### Parameter identification, model calibration and sensitivity analysis

Most parameter values were estimated directly from previously published experimental studies, prior modeling efforts [11,12] or modified slightly from literature values. These parameters and their sources are identified in Table 2. Other parameters were estimated using observed data collected from female rainbow trout during a second reproductive cycle. The latter data was previously published in [41] (oocyte diameter; plasma E2 and VTG; liver ER and VTG mRNA) or measured for this study (plasma FSH, LH, DHP and pituitary mRNA for beta subunits of FSH and LH). The data for FSH and LH was analyzed by radioimmunoassay as described by [42]. DHP was determined by enzyme immunoassay using reagents purchased from Cayman Chemical (Ann Arbor, MI). The β subunit mRNA for FSH was measured by q-RT-PCR as described in [43]. The β subunit mRNA for LH was also measured by q-RT-PCR using a similar method except the forward and reverse primers were from the LH-β gene (GenBank accession number AB050836). These primer sequences were: forward- AAACGATCCGCCTACCTGACT and reverse- AGCCACAGGGTAGGTGACATG. From these data, input curves were created using MATLAB’s cubic spline program and used to decouple the system of ODEs into smaller subsystems with fewer unknown parameters. Using the remaining input curves and the solution of the subsystem of ODEs, the error was then optimized around a least squares cost function with respect to the unknown parameters. After various decouplings, the solution for the original system of ODEs was compared to the input equations using a least squares cost function. The final cost function was then optimized by making small perturbations in all parameters not obtained explicitly through the literature. The parameters gained from this optimization process are also in Table 2. After the parameters were obtained, a global sensitivity analysis was performed using the PAWN method, outlined in [44], where the cumulative distribution functions created by a one-dimensional output distribution are used to compute density-based sensitivity indices. The model predictions of mFSH, mLH, FSH_P_, LH_Pit_, LH_P_, O_Avg_, E2, DHP, mR, R, ER, mVTG, VTG_L_, VTG_P_ and VTG_N_ using the original parameters found in Table 2 and a set of altered parameters were compared using the Euclidean norm to measure the relative changes at every hour over a 350 day time period. Summing the relative changes of the individual functions provides a one-dimensional representation of the HPOL model that normalizes the effects the altered parameters have on the individual functions. The set of altered parameters was created using a Sobol’ quasi-random sequence to obtain a uniform set of parameters that vary between ±20% of the original values listed in Table 2. For the HPOL model varying the parameters between ±20% of the original values provided a large diverse set of model predictions while lowering the proportion of similar results. These results are displayed in Table 2 with larger numbers indicating a more sensitive parameter that exerts greater influence. Many of the more sensitive parameters are those associated with the synthesis and pharmacokinetics of E2 and VTG and growth of oocytes (Table 2; highest sensitivity values). Within the pituitary, the most sensitive parameters are associated with FSH synthesis. The greater sensitivity of these parameters reflects the model’s emphasis on oocyte growth and staging. This corresponds with the biology in that oocyte growth over the entire cycle is driven by FSH through its effect on E2, which stimulates VTG production.

**Table 2:** HPOL model parameters, values and sensitivity ranking (sens.).

Parameter	Value	Units	Function	Source	Sens.
**Pituitary**					
*k*_*s*,*mFSH*_	0.05895	(pg/μg RNA)/hr	Rate of mFSH synthesis	[45]^2^^,^^6^	67
*α*_*mFSH*,*GnRH*_	2.0917	unitless	Stimulatory factor on mFSH synthesis due to GnRH	[46]^2^^,^^6^	44
*k*_*d*,*mFSH*_	0.2446	hr^-1^	Rate of mFSH degradation	Eq (7) and optimized around mFSH data.^6^	48
*k*_*s*,*mLH*_	0.0512	(pg/μg RNA)/hr	Rate of mLH synthesis	Assumed to be within ±25% of k_s,mFSH_.^6^	45
*α*_*mLH*,*GnRH*_	0.01	unitless	Stimulatory factor on mLH synthesis due to GnRH	[47]	23
*α*_*mLH*,*E*2_	4.15	ml/ng	Stimulatory factor on mLH synthesis due to E2	[45]^2^^,^^6^	56
*k*_*d*,*mLH*_	0.1046	hr^-1^	Rate of mLH degradation	Used E2 data from [41].^4^^,^^6^	66
*k*_*s*,*LH*_	1.004	(pg/μg RNA)^-1^ ng/mg pituitary/hr	Rate of LH synthesis	Used E2 data from [41] and DHP and mLH from our experiment.^4^^,^^6^	58
*k*_*d*,*LH*_	0.06	hr^-1^	Rate of LH degradation	Used E2 data from [41] and DHP and mLH from our experiment.^4^^,^^6^	46
*k*_*r*,*LH*_	66700	hr^-1^	Rate of LH release from the pituitary into the blood	Used E2 data from [41] and DHP and mLH from our experiment.^4^^,^^6^	9
*T*_*E*2,*LH*_	23	ng/ml	Threshold value for E2 levels that allow LH release from the pituitary into the plasma	[48]^2^^,^^6^	39
*n*_*E2*_	9	unitless	Determines the range of E2 levels that allow for LH release from the pituitary into the plasma	Used E2 data from [41] and DHP and mLH from our experiment.^4^^,^^6^	21
*N*_*E2*_	36.78	ml/mg pituitary	Conversion factor of E2 to the minimum amount of D2r needed to maintain the block on LH release	Used E2 data from [41] and DHP and mLH from our experiment.^4^^,^^6^	42
*N*_*DHP*_	2.8	ml/mg pituitary	Conversion factor of DHP to the minimum amount of D2r needed to maintain the block on LH release	Used E2 data from [41] and DHP and mLH from our experiment.^4^^,^^6^	22
*w*_*Pit*_	15	mg/kg	Pituitary weight	Directly measured	71
**Plasma**					
*V*_*FSH*_	156	ml/kg	FSH volume of distribution	[49,50]^2^^,^ ^6^	20
*k*_*s*,*FSH*_	0.0138	(pg/μg RNA)^-1^ ng/mg pituitary/hr	Rate of appearance of FSH in the blood	[45]^2^^,^^6^	61
*Cl*_*FSH*_	1.7372	ml/hr/kg	FSH clearance	[49]^2^^,^^6^	63
*V*_*LH*_	156	ml/kg	LH volume of distribution	Assumed to be equal to FSH, based on [49,50]	7
*Cl*_*LH*_	1.7372	ml/hr/kg	LH clearance	Assumed to be equal to FSH	14
*V*_*E*2_	261	ml/kg	E2 volume of distribution	[51]^1^	12
*Cl*_*E*2_	22	ml/hr/kg	E2 clearance	[51]^1^	75
*V*_*DHP*_	261	ml/kg	DHP volume of distribution	Assumed to be equal to E2	19
*Cl*_*DHP*_	57	ml/hr/kg	DHP clearance	Assumed from observed data.^2^^,^^6^	33
*$V_{VTG_{P}}$*	240	ml/kg	Plasma VTG volume of distribution	[52]^1^	29
*Cl*_*VTG*,*trans*_	42.2	ml/hr/kg	Intercompartmental clearance between central and peripheral	[52]^1^	26
*Cl*_*VTG*,*Seq*_	170	ml/hr/kg	Clearance of VTG from blood due to uptake by the ovaries	[12]^2^^,^^6^	31
*T*_*Seq*,*FSH*_	13.7	ng/ml	Threshold value for FSH effect on VTG uptake by the ovaries	[36]^2^^,^^6^	15
*Cl*_*VTG*_	29.3	ml/hr/kg	Total body clearance of VTG	[52]^1^	32
*$V_{VTG_{N}}$*	318	ml/kg	Volume of distribution of VTG in peripheral tissues	[52]^1^	17
**Ovary**					
*k*_*E*2_	0.0053	ng/hr/ follicle	Rate of E2 basal production	[53]^2^^,^^6^	38
*n*_*oocyte*_	2500	follicle/kg	Number of oocyte follicles per kg of fish	[38]^2^	59
*$Cl_{E2,S_{2}}$*	0.00057	ml/hr/ follicle	Clearance of E2 from the follicle due to stimulation by FSH in stage 2	Used FSH data from our experiment.^4^^,^^6^	16
*$Cl_{E2,S_{3}}$*	0.00057	ml/hr/ follicle	Clearance of E2 from the follicle due to stimulation by FSH in stage 3	Used FSH data from our experiment.^4^^,^^6^	35
*$Cl_{E2,S_{4}}$*	0.0027	ml/hr/ follicle	Clearance of E2 from the follicle due to stimulation by FSH in stage 4	Used FSH data from our experiment.^4^^,^^6^	50
*$Cl_{E2,S_{5}}$*	0.0561	ml/hr/ follicle	Clearance of E2 from the follicle due to stimulation by FSH in stage 5	Used FSH data from our experiment.^4^^,^^6^	49
*$Cl_{E2,S_{6}}$*	0.0617	ml/hr/ follicle	Clearance of E2 from the follicle due to stimulation by FSH in stage 6	Used FSH data from our experiment.^4^^,^^6^	28
*$Cl_{DHP,S_{2}}$*	0.0003	ml/hr/ follicle	Clearance of DHP from the follicle due to stimulation by LH in stage 2	Used LH data from our experiment.^4^^,^^6^	11
*$Cl_{DHP,S_{3}}$*	0.0003	ml/hr/ follicle	Clearance of DHP from the follicle due to stimulation by LH in stage 3	Used LH data from our experiment.^4^^,^^6^	24
*$Cl_{DHP,S_{4}}$*	0.0003	ml/hr/ follicle	Clearance of DHP from the follicle due to stimulation by LH in stage 4	Used LH data from our experiment.^4^^,^^6^	18
*$Cl_{DHP,S_{5}}$*	0.0003	ml/hr/ follicle	Clearance of DHP from the follicle due to stimulation by LH in stage 5	Used LH data from our experiment.^4^^,^^6^	27
*$Cl_{DHP,S_{6}}$*	0.3	ml/hr/ follicle	Clearance of DHP from the follicle due to stimulation by LH in stage 6	Used LH data from our experiment.^4^^,^^6^	4
*$Cl_{DHP,S_{FOM}}$*	0.79	ml/hr/ follicle	Clearance of DHP from the follicle due to stimulation by LH in FOM	Used LH data from our experiment.^4^^,^^6^	3
*_$k_{NV,O_{Avg}}$_*	0.00067	mm/hr/kg	Growth rate of individual non-vitellogenic follicles	[53]^2^^,^^6^	55
*_$k_{V,O_{Avg}}$_*	2.19e-7	mm/mg	Growth rate per amount of VTG sequestered by an individual follicle	Used VTG data from [45] and FSH data from our experiment.^4^^,^^6^	25
*_$\alpha_{O_{Var},S_{1}}$_*	0.0214	mm^2^	Maximal variance of follicles in stage 1	[38]^2^^,^^6^	5
*$\alpha_{O_{Var},S_{2}}$*	0.0433	mm^2^	Maximal variance of follicles in stage 2	[38] ^2^^,^^6^	6
*$\alpha_{O_{Var},S_{3}}$*	0.0748	mm^2^	Maximal variance of follicles in stage 3	[38] ^2^^,^^6^	40
*$\alpha_{O_{Var},S_{4}}$*	0.0907	mm^2^	Maximal variance of follicles in stage 4	[38] ^2^^,^^6^	57
*$\alpha_{O_{Var},S_{5}}$*	0.0338	mm^2^	Maximal variance of follicles in stage 5	[38] ^2^^,^^6^	36
*$\alpha_{O_{Var},S_{6}}$*	0.014	mm^2^	Maximal variance of follicles in stage 6	[38] ^2^^,^^6^	2
*$\alpha_{O_{Var},S_{FOM}}$*	0.0067	mm^2^	Maximal variance of the follicles in FOM	[38] ^2^^,^^6^	8
*s*_*1*_	0.2	mm	Follicle diameter dividing stages 1 and 2 (follicles are able to produce E2)	[5]^1^	13
*s*_*2*_	0.6	mm	Follicle diameter dividing stages 2 and 3 (follicles are able to take up VTG)	[37]^1^	68
*s*_*3*_	1.17	mm	Follicle diameter dividing stages 3 and 4 (follicles enter the mid-vitellogenic stage)	Used the data from our experiment and [41].^6^	69
*s*_*4*_	1.69	mm	Follicle diameter dividing stages 4 and 5 (follicles enter the late vitellogenic stages)	Used the data from our experiment and [41].^6^	74
*s*_*5*_	3.4	mm	Follicle diameter dividing stages 5 and 6 (follicles are able to produce substantially more DHP)	Used the data from our experiment and [41].^6^	62
*s*_*6*_	5.3	mm	Follicle diameter dividing stages 6 and FOM (follicles are no longer able to sequester VTG)	Based on [5]: (s_6_-s_2_)/O_M_ = 0.84, where O_M_ is the maximum average oocyte diameter from [41]	10
*FOM*_*final*_	0.98	unitless	Proportion of follicles in FOM needed before ovulation	Assumed^6^	30
*DHP*_*final*_	120	ng/ml	Concentration of DHP needed before ovulation can occur	Assumed based on DHP levels from present experiment.^6^	1
**Liver**					
*w*_*L*_	15	g/kg	Liver weight	[12]^3^	41
*k*_*s*,*mR*_	30	pg/ug RNA/hr	Rate of mR synthesis	[12]^3^	78
*α*_*mR*,*ER*_	0.0667	g liver/ fmol	Maximal stimulatory fold-increase in mR synthesis due to ER complex	[12]^3^	65
*k*_*d*,*mR*_	0.5	hr^-1^	Rate of mR degradation	[12]^3^	73
*k*_*s*,*R*_	0.0113	(pg/ug RNA)^-1^ fmol/g liver/hr	Rate of R synthesis	[12]^3^	76
*k*_*d*,*R*_	0.466	hr^-1^	Rate of R degradation	[12]^3^	52
*k*_*on*,*ER*_	0.826	(ng/ml)^-1^ hr^-1^	Association rate constant for ER	[12]^3^	60
*k*_*off*,*ER*_	0.347	hr^-1^	Dissociation rate constant for ER	[12]^3^	64
*k*_*d*,*ER*_	0.0766	hr^-1^	Rate of ER complex degradation	[12]^3^	77
*k*_*s*,*mVTG*_	6.93E-05	pg/ug RNA/hr	Rate of mVTG synthesis	[12]^3^	70
*α*_*mVTG*,*ER*_	5.456e6	g liver/ fmol	Stimulatory factor on VTG synthesis due to ER complex	[12]^3^	72
*k*_*d*,*mVTG*_	0.00462	hr^-1^	Rate of mVTG degradation	[12]^3^	54
*k*_*s*,*VTG*_	9.02E-06	mg/g liver/hr	Rate of VTG synthesis	[12]^2^^,^^6^	53
*N*_*mVTG*_	1000	pg/ug RNA	Scaling factor for mVTG concentration	[12]^3^	47
*γ*	2.48	unitless	Amplification factor on the translation of VTG	[12]^3^	79
*k*_*r*,*VTG*_	7.87	hr^-1^	Rate of VTG release from the liver into the plasma	[12]^3^	43
**Transit Compartments**					
*D*_*FSH*,*E2*_	875	hr	Delay of E2 synthesis due to FSH	Used FSH data from our experiment and E2 data from [41].^5^^,^^6^	51
*D*_*E2*,*mLH*_	240	hr	Delay of mLH synthesis due to E2	Used E2 data from [41] and mLH data from our experiment.^5^^,^^6^	37
*D*_*LH*,*DHP*_	72	hr	Delay of DHP synthesis due to LH	Used LH data and DHP data from our experiment.^5^^,^^6^	34

^1^ Parameter value was obtained directly from source.

^2^ Guided initial estimates of the parameter value.

^3^ Parameter value was obtained from prior modeling efforts.

^4^ Specified data was used to create an input curve and an initial estimate was obtained through optimization methods solving a minimum number of differential equations.

^5^ Initial estimate was obtained by calculating time delays in the relative extrema from specified data.

^6^ Final parameter value was refined through optimization methods using experimental data from [41] and our experiment. See the Methods Section for details.

## Results

**Fig 2:**
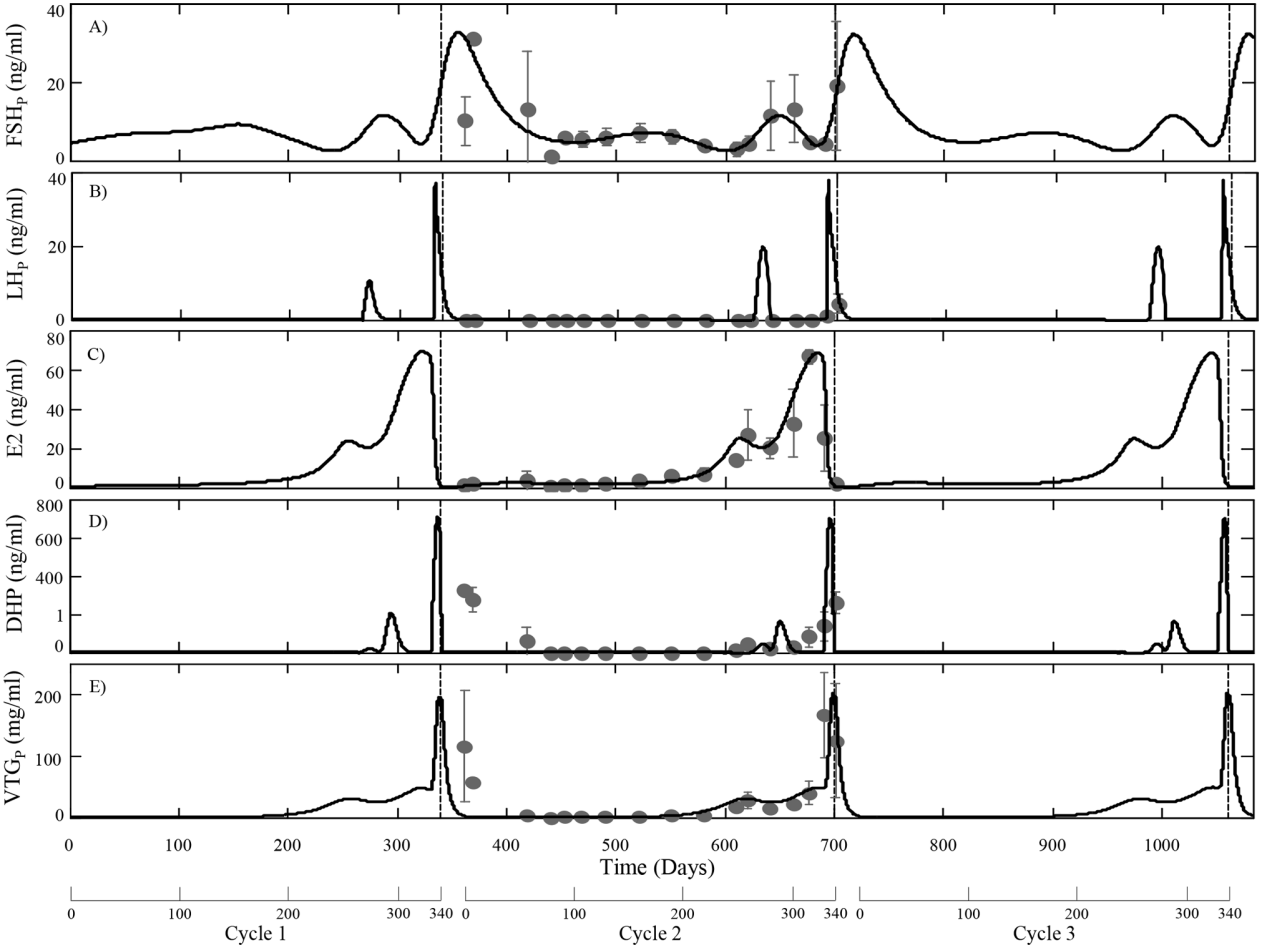
HPOL model predictions for circulating levels of FSH (A), LH (B), E2 (C), DHP (D) and VTG (E) over three reproductive cycles (approximately three years) using the parameters in Table 2. Observed data (mean ± SD, *n* = 3) measured from a cohort of second time spawning female trout is shown as dark grey circles. The observed data for FSH, LH and DHP was measured as part of this study or from [45]. Three weeks after spawning average oocyte growth was reset to O_Avg_(0). Circulating DHP is shown on a nonlinear scale to emphasize low levels (less than 1 ng/ml) of DHP before FOM.

**Fig 3:**
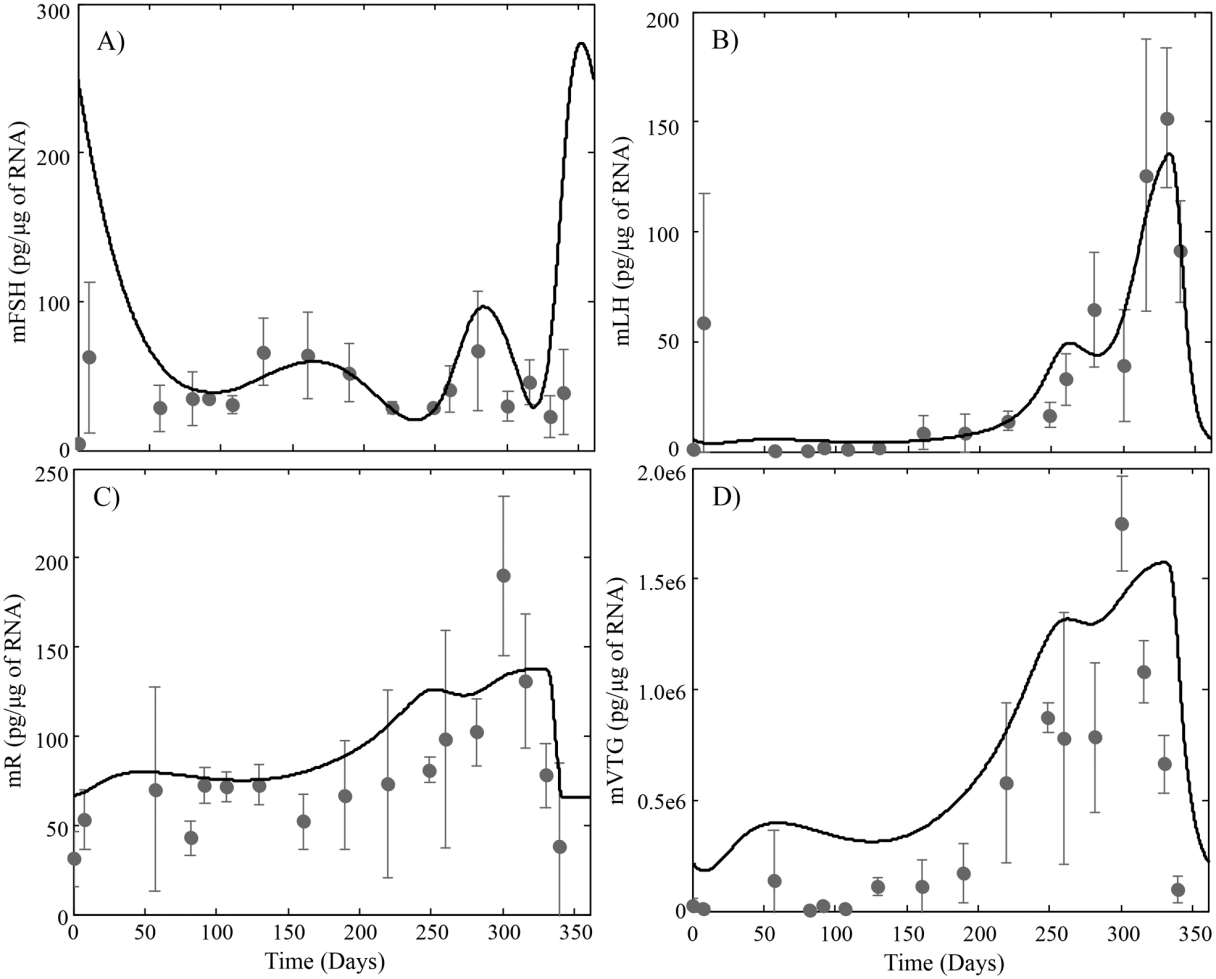
HPOL model predictions for (A) pituitary levels of FSH_β_ subunit mRNA, (B) pituitary levels of LH_β_ subunit mRNA, (C) Hepatic levels of E2 receptor mRNA and (D) Hepatic levels of VTG mRNA using the parameters in Table 2. Observed data (dark grey circles; mean ±TG mR*n* = 3) was measured from the same individuals used for Fig 2.

**Fig 4:**
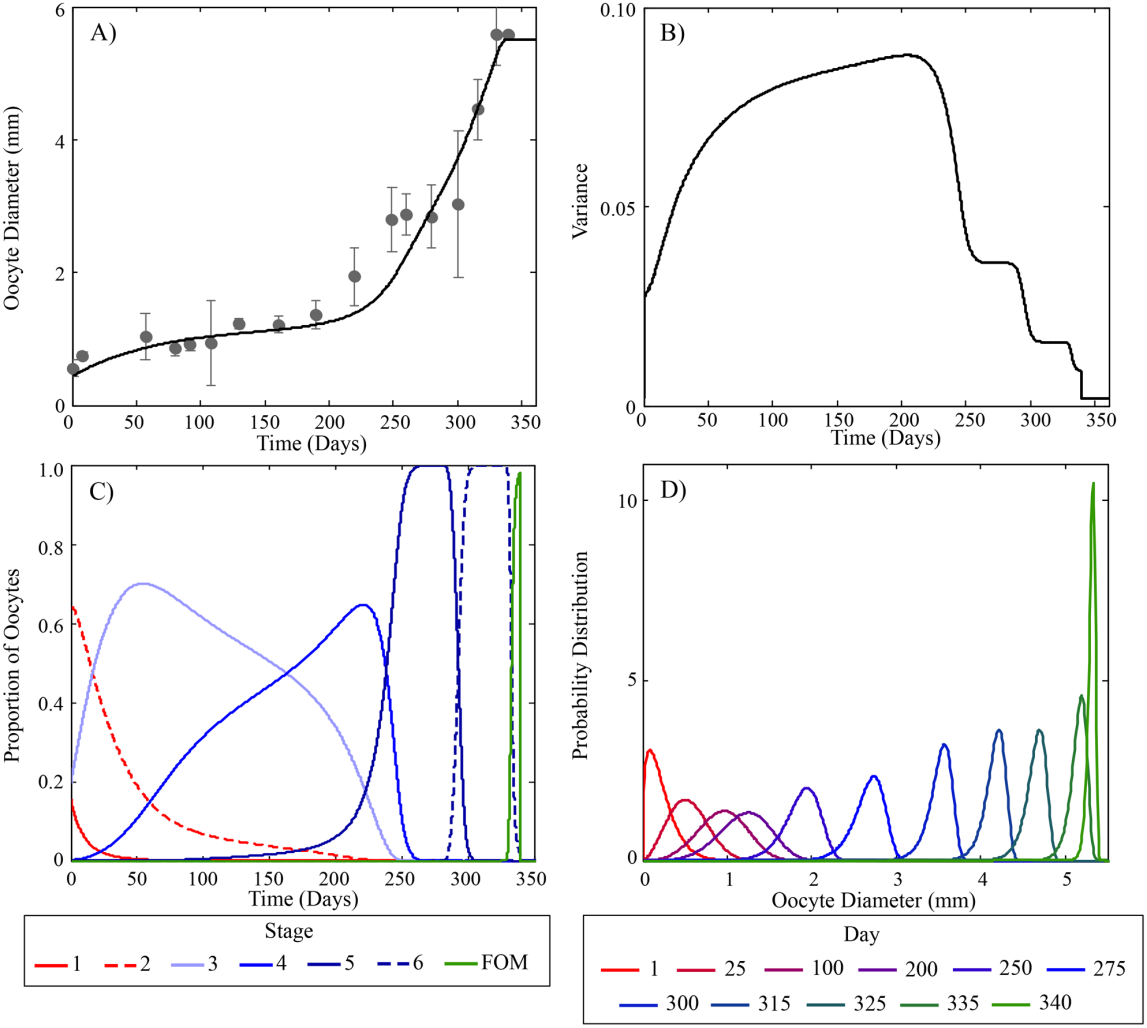
Predicted oocyte growth and staging during a second reproductive cycle in rainbow trout. (A) Predicted average oocyte growth and experimentally measured values [41]. Measured values were determined from the same individuals used for Figs 2 and 3. (B) Model predicted variance of oocyte diameters. (C) Model predicted proportion and duration of oocyte stages during a second reproductive cycle. Solid and dashed red lines are pre-vitellogenic stages (1 and 2), light to dark blue lines (stages 3–6) are early, mid and late vitellogenic stages and green line is the proportion of oocytes at FOM. (D) Model predictions of the distribution of oocyte diameter at various times during the reproductive cycle. Note the variability of oocyte diameter rapidly increases at the beginning of the cycle and decreases prior to ovulation.

The lifespan of rainbow trout in their natural habitat is quite variable and may last from three to seven years [54]. Most populations of rainbow trout mature in their second or third year and may spawn in successive years up to five times, typically in late fall or early spring [55,56,57]. A complete reproductive cycle in female rainbow trout normally requires 345–365 days, which is reproduced in the model. We’ve chosen to focus on changes in model variables during a second reproductive cycle because a large portion of the experimental data that was used to guide model development was collected during this time period. Model simulations were performed using ode23s in MATLAB (version R2014a) and the parameter values are listed in Table 2. A summary of model predictions for tissue specific and circulating variables are presented in Figs 2–4. Included in these figures are experimentally measured values from a cohort of females sampled during a second reproductive cycle. Model simulations were compared to this data set as it represents, to our knowledge, the most complete set of measurements of model variables in a single cohort of female rainbow trout. Although portions of this data set were used to guide estimation for some parameters (notably synthesis rates of mFSH and mLH), many others were determined from separate in vivo and in vitro studies (Table 2) and the model was not optimized or fitted to this data set. The model is also capable of continuous predictions, which we demonstrate for circulating variables (FSH, LH, E2, DHP and VTG) over three successive spawning cycles (Fig 2).

Inspection of model predicted and observed values in Figs 2 and 3 suggest the model adequately describes the changes that occur during the reproductive cycle. Model predicted levels of circulating FSH reflect the pattern of mFSH synthesis in the pituitary throughout the cycle and is in close agreement with observed data (Figs 2A and 3A). After reaching the ovaries, FSH stimulates the follicles to produce E2 (Fig 2C), the rate of which is stage dependent with increasing rates until FOM (Table 2). Hence, the relative extrema of circulating E2 levels reflect the relative extrema in circulating levels of FSH after an approximately 36 day delay. This activity of FSH is known to be mediated by a membrane bound receptor in the thecal and granulosa cells of the follicle [1]. We have not included GTH receptor dynamics in this version of the model and instead describe the stimulation of E2 synthesis with a single rate parameter. Although not modeled explicitly, we assume that a portion of the stage specific changes in the rate of E2 synthesis is associated with changes in expression of the FSH receptor. When circulating levels of E2 increase beyond a threshold, subsequent declines trigger the release of LH. The model predicts a premature rise in circulating LH that occurs approximately 80 days before the main surge in LH that triggers the final changes leading to ovulation (Fig 2B). This early or premature rise in LH is not reflected in the observed data although it may have been easily missed due to the rapid increase and decline of LH in blood. One possible role for an early LH increase is to stimulate DHP synthesis, which has been suggested to promote meiosis in early oogonia that will become oocytes during the next cycle [58]. The model over predicts mVTG synthesis in the liver (Fig 3D) although prediction of circulating VTG closely matches observed data (Fig 2E).

As the oocytes progress into stage 6 and FOM, rising DHP levels eventually pass a threshold and fulfill one of two requirements for ovulation. The other requirement for ovulation depends on the proportion of oocytes that reach FOM, which in the model depends on the growth and variance of the oocytes (Fig 4A and 4B respectively). Initially, the distribution of oocytes is positively skewed with little variation in size. As vitellogenesis begins the growth rate of individual oocytes varies depending on a number of factors causing a large variation in sizes; the peak of which occurs during mid-vitellogenesis, stage 4. When the oocytes enter the post-vitellogenic phases and near FOM, the growth rate becomes inversely related to size causing a reduction in variation and the distribution to be negatively skewed. This gradual increase and then steep decline in oocyte variance (as seen in Fig 4D) causes the early and mid vitellogenic stages to be the largest growth stages (Fig 4C). Subsequent rapid growth of oocytes during stages 5 and 6, days 225 through 335, is caused by the steep increase in VTG production (Fig 4A). When the oocytes enter FOM they lose the capacity to sequester VTG causing a transient spike in circulating VTG levels (Fig 2E).

**Fig 5:**
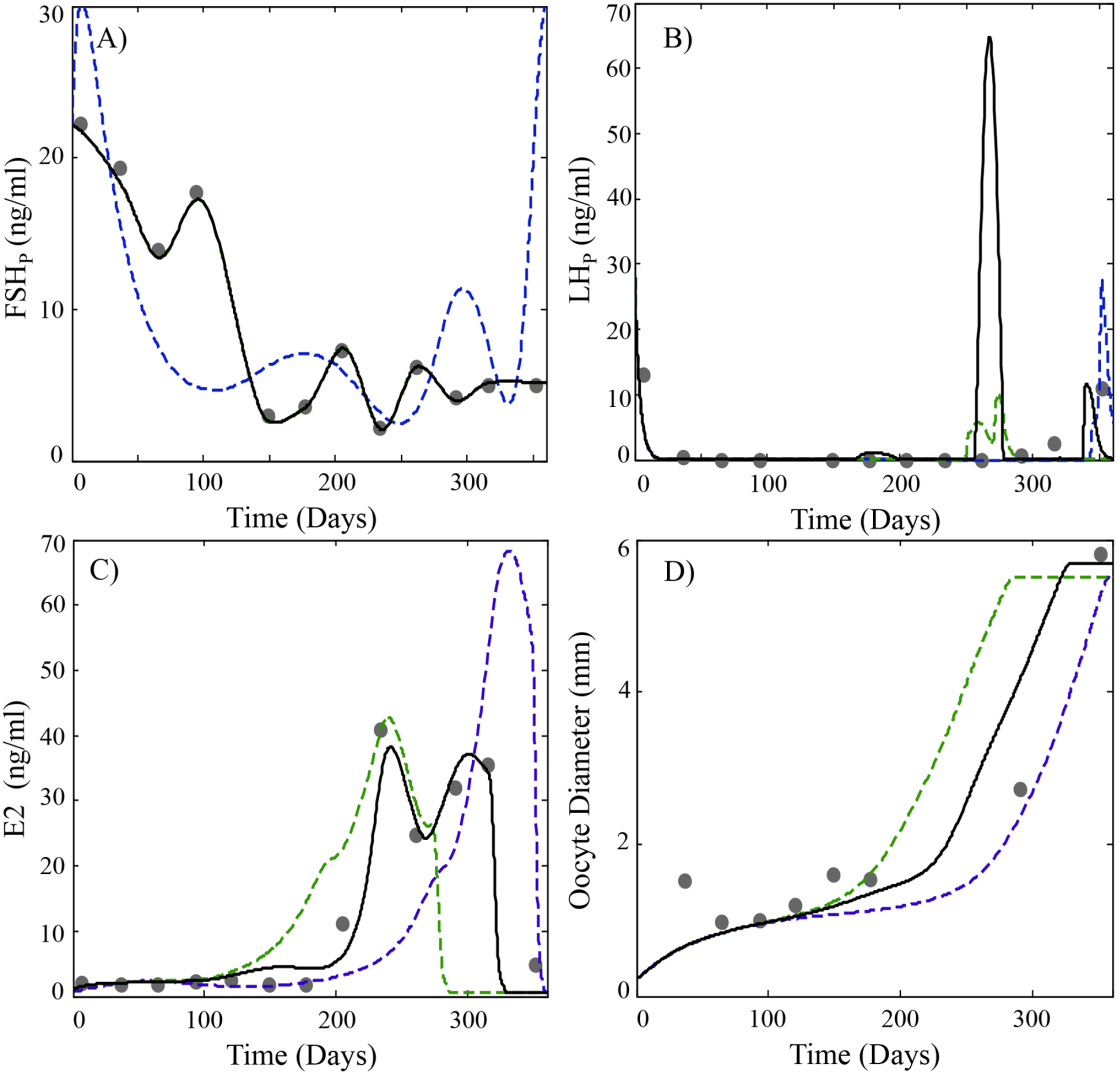
Predicted plasma profiles of (A) FSH, (B) LH, (C) E2 and (D) average oocyte diameter (O_Avg_) compared to experimental data (black circles) obtained from [59]. Model simulations using parameter estimates listed in Table 2 and the same GnRH function used in Figs 2–4 are shown as dashed blue lines. Simulations using a customized GnRH function based on measured FSH levels in [59] (see also Appendix S5) are shown as a green dashed line. Simulations with the customized GnRH function and altered vitellogenic staging parameters (s_3_ = 1.19, s_4_ = 1.74, s_5_ = 3.51 and s_6_ = 5.48) are shown as a black solid line. A regression curve was used to map the GSI data in [59] to the average oocyte diameter (See Appendix S1).

**Fig 6:**
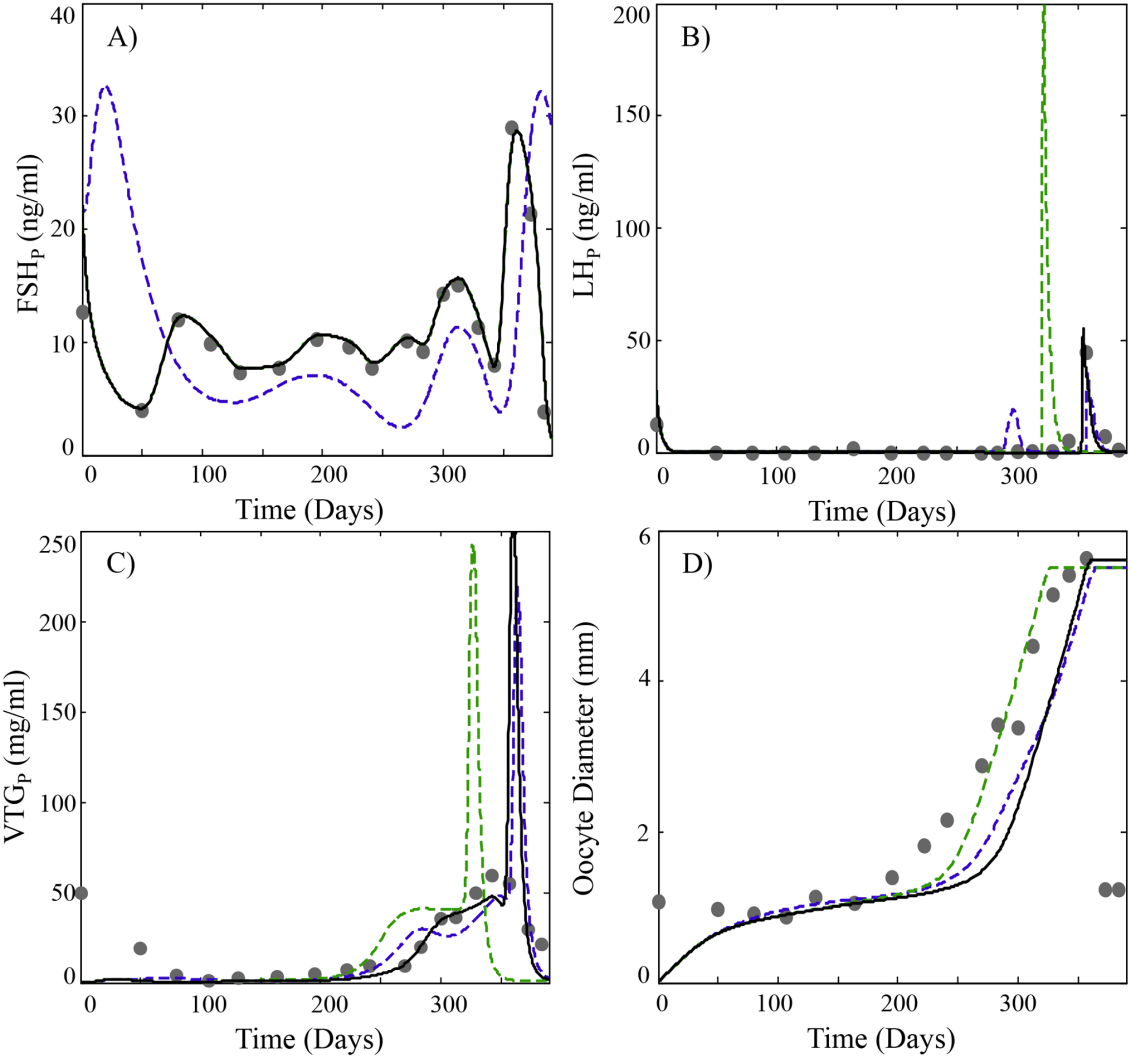
Predicted plasma profiles of (A) FSH, (B) LH, (C) E2 and (D) average oocyte diameter (O_Avg_) compared to experimental data (black circles) obtained from [53,60]. Model simulations using parameter estimates listed in Table 2 and the same GnRH function used in Figs 2–4 are shown as dashed blue lines. Simulations using a customized GnRH function based on measured FSH levels in [53,60] (see also Appendix S5) are shown as a green dashed line. Simulations with the customized GnRH function and altered vitellogenic staging parameters (s_3_ = 1.21, s_4_ = 1.76, s_5_ = 3.48 and s_6_ = 5.39) are shown as a black solid line. A regression curve was used to map the GSI data in [53] to the average oocyte diameter (See Appendix S1).

Because parameterization of the model relied on multiple in vitro and in vivo sources of experimental data, it is reasonable to expect the model to be relatively robust in describing reproductive performance beyond the data set used for initial validation (Figs 2–4). To demonstrate this, we present two examples using experimental data from three additional sources. These studies monitored several model variables (FSH, LH, VTG, E2) in cohorts of second-time spawning rainbow trout [53,59,60]. Comparison of HPOL model predictions with observed data from these studies is shown on Figs 5 and 6. In both examples, subtle differences in observed and predicted FSH levels lead to differences in either E2 (Fig 5; [59]) or VTG (Fig 6; [60]). However, the model is still able to reasonably describe oocyte growth patterns from both studies (Figs 5 and 6). If the model’s GnRH function is optimized for the FSH profiles for each study, an improvement in the prediction of E2 and VTG is observed (green lines on Figs 5 and 6). By adjusting the oocyte sizes that define stages 3–6 to account for approximately 10%, 9%, 31% and 34% of oocyte growth (black lines on Figs 5 and 6), model predictions of oocyte growth more closely match the observed values. These adjustments made to the oocyte stages follow a similar logic to the derivation of the parameter dividing stage 6 from FOM, s_6_, in Table 2 and uses the parameter values listed in Table 2 to approximate the percentage of growth each of the vitellogenic stages should account for. These examples highlight the ability of the model to predict reproductive variables in rainbow trout but also expose some limitations of broadly using the model with rainbow trout as currently parameterized. There is considerable plasticity in spawning behavior among rainbow trout, which may spawn at various times throughout the year [57]. Thus, it should be expected that some natural variability in GnRH and FSH synthesis will occur in different fish populations and this variation will introduce changes in model variables associated with E2 and VTG synthesis. However, the generally good agreement of model simulations with previously published data supports validation of the model and the parameters gained from optimization in Table 2 and when necessary, should require only minor adjustments to describe reproduction in different populations of rainbow trout.

## Discussion

Reproduction in fishes and other vertebrates represents the timely coordination of many endocrine factors that culminate in the production of mature, viable gametes. The model presented here accurately describes the essential processes of GTH production, oocyte growth, vitellogenesis and final maturation in a group synchronous annual spawning fish. The model is an improvement over previous efforts in salmonids, which focused more narrowly on either GTH and steroid production [11] or on vitellogenesis [12]. Here we have combined and expanded these models into a single version that can continuously describe reproduction in trout over successive cycles. The present model provides a description of GnRH production and its stimulation of GTH synthesis throughout the reproductive cycle. This is in contrast to [11] where GnRH was modeled at a constant level of 50 arbitrary units between days 75 and 250 of the reproductive cycle. This simplified approach proved inadequate to capture the seasonal changes observed for pituitary expression of mFSH and secretion of FSH into plasma. Accurate prediction of mFSH expression is particularly important due to the tight coupling with circulating levels of FSH (Figs 2A and 3A e.g relative changes in pituitary levels of FSH_*b*_ coincide with changes in FSH) and the key role FSH performs in regulating E2 production. This becomes evident in the simulations shown in Figs 5 and 6 and how small adjustments in the GnRH function to better describe FSH improved predictions of E2 and VTG.

Another specific improvement in the present model is the more extensive description of oocyte staging and oocyte growth. The model explicitly describes oocyte size, which is used to define growth and to differentiate the developmental stages of maturation. Including distinct stages of development is advantageous because it is a traditional measurement reported by fish biologists and many processes such as VTG accumulation are stage dependent. Also, within the maturing trout ovary there is evidence for sub-populations of oocytes with overlapping developmental stages and associated stage dependent growth rates [38]. Defining the stages provides the ability to include attributes that are stage specific and allow for overlap. The majority of oocytes will fall into one stage category at a time, allowing for segregation of stages. However, segregated staging may cause discontinuities in the ODE system and would not satisfy the criteria for uniqueness and existence of solutions for ODEs, the Picard-Lindelöf theorem [61]. Although smooth approximations could be used to define the staging to avoid discontinuities, the solution would be irregular (See Appendix S4). An added attraction of overlapping staging is that it narrows the distinction made between synchronous and asynchronous spawning fish and offers the potential for future studies to explore control processes that contribute to the evolution of reproductive strategies.

A challenging aspect of HPOL model development is how to approach the timely release of LH from the pituitary into the bloodstream near the end of the reproductive cycle. In constructing the model, we evaluated several equations of varying complexity for describing the release of LH (see Appendix S2). The release primarily depends on declining E2 levels that typically occur during the later stages of oocyte development. Hence, LH release could simply be restricted to occur when E2 declines during a narrowly defined time period such as during late vitellogenesis and FOM:

$$R_{LH1}(t)=\left(S_{6}(t)+S_{FOM}(t)\right)\cdot H_{-}\left(\left[E2\right],T_{E2,LH},n_{E2,LH}\right)\left[LH_{Pit}\right]$$ (28)

This approach is similar to the release function used in [11]. However, there are reports of elevated blood levels of LH before the final decline of E2 [41,59,62] and other evidence suggesting the release of LH is not confined to the final stages of reproduction [33]. Therefore, a more biologically realistic approach would be to remove restrictions on stage specificity and allow LH release to occur when E2 levels are declining faster than a threshold (T_E2_)_._ Another consideration is whether secretion of LH from the pituitary can simply be treated as a bolus release (e.g. near instantaneous), which has the effect of causing a large spike in predicted blood levels of LH. Although adequate to describe the LH plasma profile, this also tends to overestimate peak levels of LH because the actual release probably occurs as a more gradual process. It also ignores the role that dopamine plays in maintaining the block on LH release. This led us to consider the release of LH as the spillover of unblocked LH in the pituitary, Eq (12). Alternatively, a more physiologically explicit description of dopamine signaling and levels of D2r could be added to the model. However, we have chosen not to add this to the model at this time because of the added complexity and lack of experimental data in trout to validate model predictions.

Another important aspect of model development was to incorporate time delays associated with biological processes not specifically accounted for in the model. An example of this is the receptor-mediated effects of FSH on the follicle, particularly with respect to steroidogenesis, which occur over an extended time period. Rather than attempt to include additional parameters to characterize FSH activity, we chose to use transit compartments to represent time delays that are due to receptor signaling, gene expression and associated changes in biochemical processes. An alternative to transit compartments would be the use of delayed differential equations (DDEs). The advantages of DDEs reside with the potential to describe more complex behavior and improve accuracy with less parameters [24]. However, the use of DDEs requires knowledge of a variable’s behavior before the initial time point, information which may not be available or difficult to ascertain during initial ovarian recrudescence. Transit compartments are a simple ODE approximation of DDEs which only require knowledge of present conditions and are a well-established tool in pharmacokinetic-pharmacodynamic modeling to characterize time delays [25]. Furthermore, transit compartments can be readily exchanged with sub-models to provide a more detailed approach to specific biological processes. For example, in the present model E2 is synthesized and added to the bloodstream after FSH enters the ovaries with a time delay of D_E2,FSH_ hours. A more detailed model of the synthesis of E2 could be used such as the type described by [18] and inserted in place of the transit compartments between FSH and E2. Conversely, the present model could be simplified by inserting transit compartments between E2 and plasma VTG to approximate plasma level of VTG (see Appendix S3 for results):

$$\frac{d}{dt}\left[VTG_{P}\right]=\frac{1}{V_{VTG_{P}}}\left(k_{E2,VTG}\left[E2^{TC_{n}}\right]+Cl_{VTG,trans}\left[VTG_{N}\right]-\left(Cl_{VTG,trans}+Seq(t)+Cl_{VTG}\right)\left[VTG_{P}\right]\right).$$ (29)

This latter approach might be used when the model is being applied to other fish species that lack detailed experimental data on vitellogenesis. In either case, the use of transit compartments provides flexibility for increasing or decreasing complexity as needed.

### Application and future work

**Fig 7:**
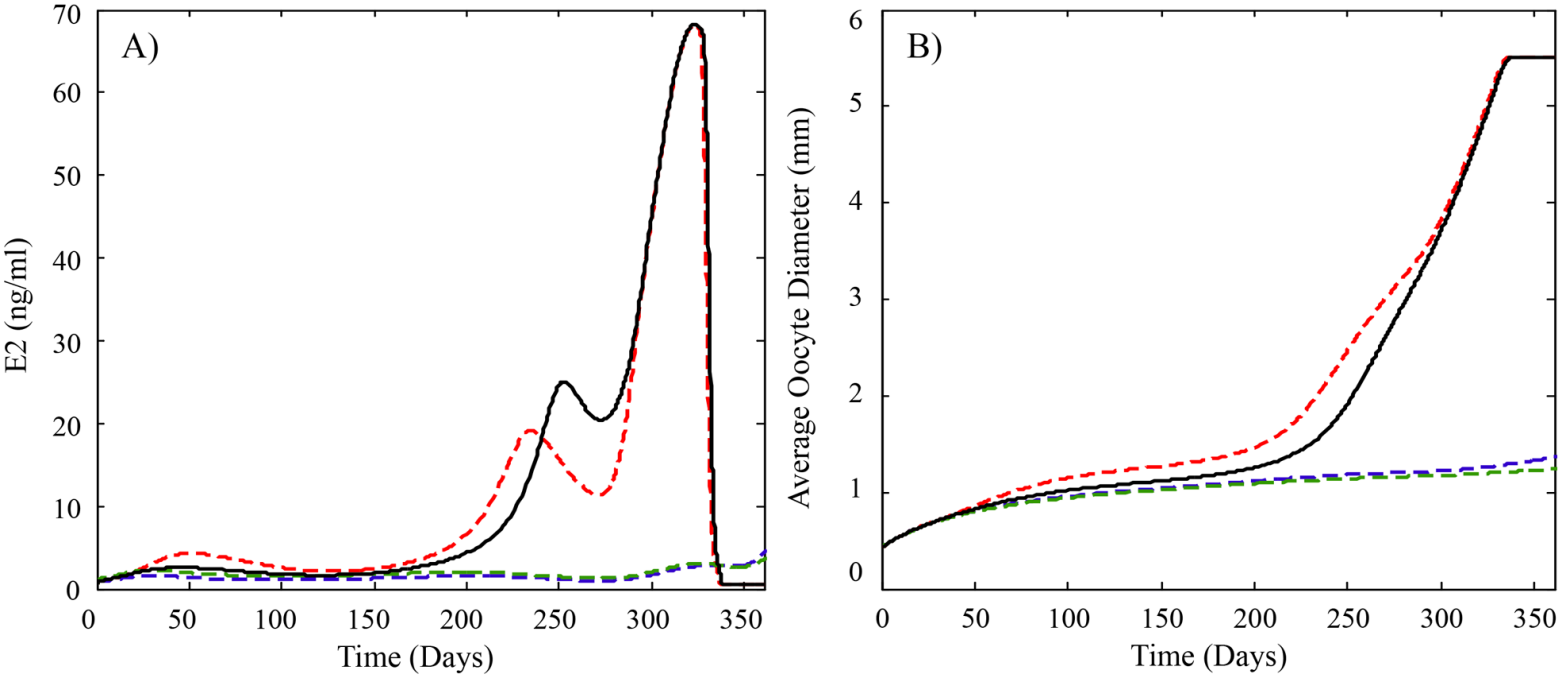
Model predicted profiles of plasma levels of (A) E2 and (B) average oocyte growth for rainbow trout exposed to trenbolone. Previously published studies suggest trenbolone exposure may decrease E2 synthesis to varying degrees depending on exposure levels, and also increase the clearance of E2 from plasma and increase the secretion of FSH in trout. The solid black line is the model predictions for fish not exposed to trenbolone and uses the parameter values found in Table 2. The dashed lines depict model predictions of known effects of trout that are exposed to trenbolone and use parameter values found in Table 2 except for Cl_E2,Sj_ = 0.54*Cl_E2,Sj_ for j = 4 and 5, k_s,FSH_ = 4*k_s,FSH_ during Stages 1 and 2. The blue dashed line also uses Cl_E2_ = 1.41*Cl_E2_ and the green dashed line uses k_s,mVTG_ = 0.6*k_s,mVTG_. The model predicts that ovulation is unlikely to occur when an increase in E2 clearance and/or a decrease in mVTG synthesis is added to the effects because the delay in ovulation is more than 50 days.

An important value of quantitative biological models is the ability to perform simulations that vary model parameters in ways that are associated with various physiological conditions, real or hypothetical. In this manner, the rainbow trout HPOL model can be a tool to explore the effects of diverse physical and chemical stressors on reproduction. As an example, we consider trenbolone exposure to female rainbow trout. Trenbolone is a synthetic testosterone analogue that is used to promote growth in cattle. Concerns over the possible release of trenbolone to aquatic environments have prompted several studies on its effects to fish. In fathead minnows, trenbolone exposures (27 ng/L and higher) for up to three weeks while fish are actively spawning, caused a rapid and significant decline in circulating E2 and VTG levels and eventual cessation of spawning [51,63,64]. Subsequent in vitro experiments with fathead minnow ovary tissue suggest the decrease was due in part to decreased expression of enzymes involved in E2 synthesis [65]. In short-term exposures (one–four weeks) to female rainbow trout, trenbolone exposure stimulated FSH release in pre-vitellogenic fish, but also caused decreases in E2 levels in both pre- and mid-vitellogenic trout [51]. Other experiments with trout indicated plasma clearance of E2 was increased by 50% [51]. We were interested in estimating whether these effects in rainbow trout alone or in various combinations would be sufficient to prevent reproduction if trout were assumed to be continuously exposed throughout the reproductive cycle. We adjusted the following parameters based on the prior experimental evidence: FSH release (*k*_*s*,*FSH*_; increased 4x), E2 synthesis (SE2O4-5; decreased 46%), E2 plasma clearance (*Cl*_*E2*_; increased 41%) and VTG expression (*k*_*s*,*mVTG*_; decreased 40%). A summary of model simulations for E2 and oocyte diameter is shown in Fig 7. Increasing the release of FSH alone or in combination with up to 46% decreases in E2 synthesis did not affect overall oocyte growth (Fig 7). If a decrease in VTG expression or increased E2 clearance occurs simultaneously with decreased E2 synthesis, then oocyte growth is slowed to an extent that ovulation would be unlikely to occur (Fig 7). These simulations suggest trenbolone effects on E2 synthesis by itself are not sufficient to cause reproductive failure in trout and additional effects occurring simultaneously elsewhere in the system are needed. Additional experimental research is needed to verify these observations but the exercise illustrates the potential for the HPOL model to incorporate in vivo and in vitro results for predictions of reproductive performance. This is a valuable attribute because long-term or complete reproductive cycle exposures are costly to perform and require many animals.

An emerging concept in environmental health research is the adverse outcome pathway (AOP) [66]. An AOP seeks to extrapolate effects of stressors measured at the cellular / sub-cellular level of biological organization to whole organism and population-level effects. It is anticipated that future research efforts will increasingly rely on in vitro methods to generate health effects data. Thus, mathematical models such as the trout HPOL model presented here will be useful for translating in vitro based observations into whole fish effects. As we consider future efforts to develop the next-generation HPOL model, emphasis will be placed on linking model outputs more closely to the types of input data used for population models. This would include expanding the description of oocyte development to provide more detail on egg quality and lifetime fecundity of rainbow trout over multiple spawning cycles.

## Supporting Information

S1 Fig Predicted plasma levels of LH using different release functions over an entire reproductive cycle, (A), and from day 250 until the end of the reproductive cycle, (B). The black dashed line is using the final release function, R_LH_, given by Eq (14). The red line is using the release function R_LH1_ given by Eq (28). The blue line is using the release function R_LH2_ given by Equation (S5). The green line is using the release function R_LH3_ given by Equation (S7). Plasma LH data from second time spawning female rainbow trout, also used in Fig 2, is represented by the black circles. While the release functions mathematically give similar results they are lacking in biological accuracy.
(TIF)

S2 Fig Model predictions for circulating VTG (A) and average oocyte growth (B) showing the transit compartments ability to predict the VTG sub-model. The black dashed line uses the complete VTG model, Eq (21) through Eq (27), to describe E2’s effects on VTG. The blue solid line uses transit compartments to approximate the effects E2 has on VTG using delay of 100 hours.
(TIF)

S3 Fig E2 plasma levels (A) and average oocyte growth (B) with and without the overlapping developmental stages. The dashed black line assumes that at any point in time the oocytes could be divided into multiple developmental stages. The blue solid line assumes the developmental stages are segregated.
(TIF)

S1 Appendices This is a.docx file containing appendices 1–5. (DOCX)

S1 Code The file is the model code. (M)

S2 Code The file is a docx version of the model code. (DOCX)

S1 Text Supporting Information. The file contains parameter values used with the model code files.
(XLSX)

S2 Text Supporting Information. The file contains observed data used in Figs 2–5.
(XLS)

S3 Text Supporting Information. The file contains GnRH values used with the simulations presented in Figs 2–5.
(XLSX)
