# Supplementary figures and images for: A Computational Model of the Rainbow Trout Hypothalamus-Pituitary-Ovary-Liver Axis
Source: PLoS Comput Biol. 2016 Apr 20;12(4):e1004874. doi: 10.1371/journal.pcbi.1004874 (PMC4838294; doi:10.1371/journal.pcbi.1004874)

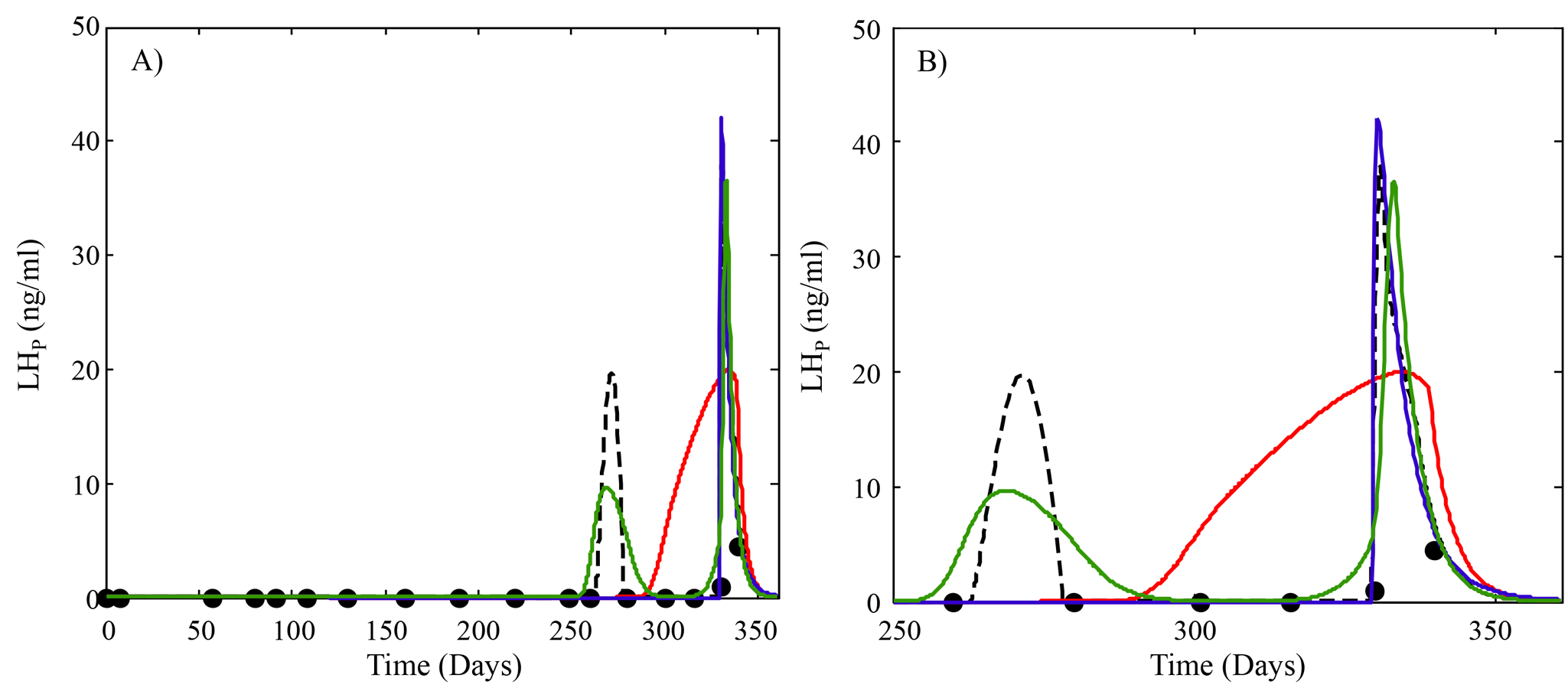

Supplement: S1 Fig — The black dashed line is using the final release function, RLH, given by Eq (14). The red line is using the release function RLH1 given by Eq (28). The blue line is using the release function RLH2 given by Equation (S5). The green line is using the release function RLH3 given by Equation (S7). Plasma LH data from second time spawning female rainbow trout, also used in Fig 2, is represented by the black circles. While the release functions mathematically give similar results they are lacking in biological accuracy. (TIF) [file pcbi.1004874.s001.tif]

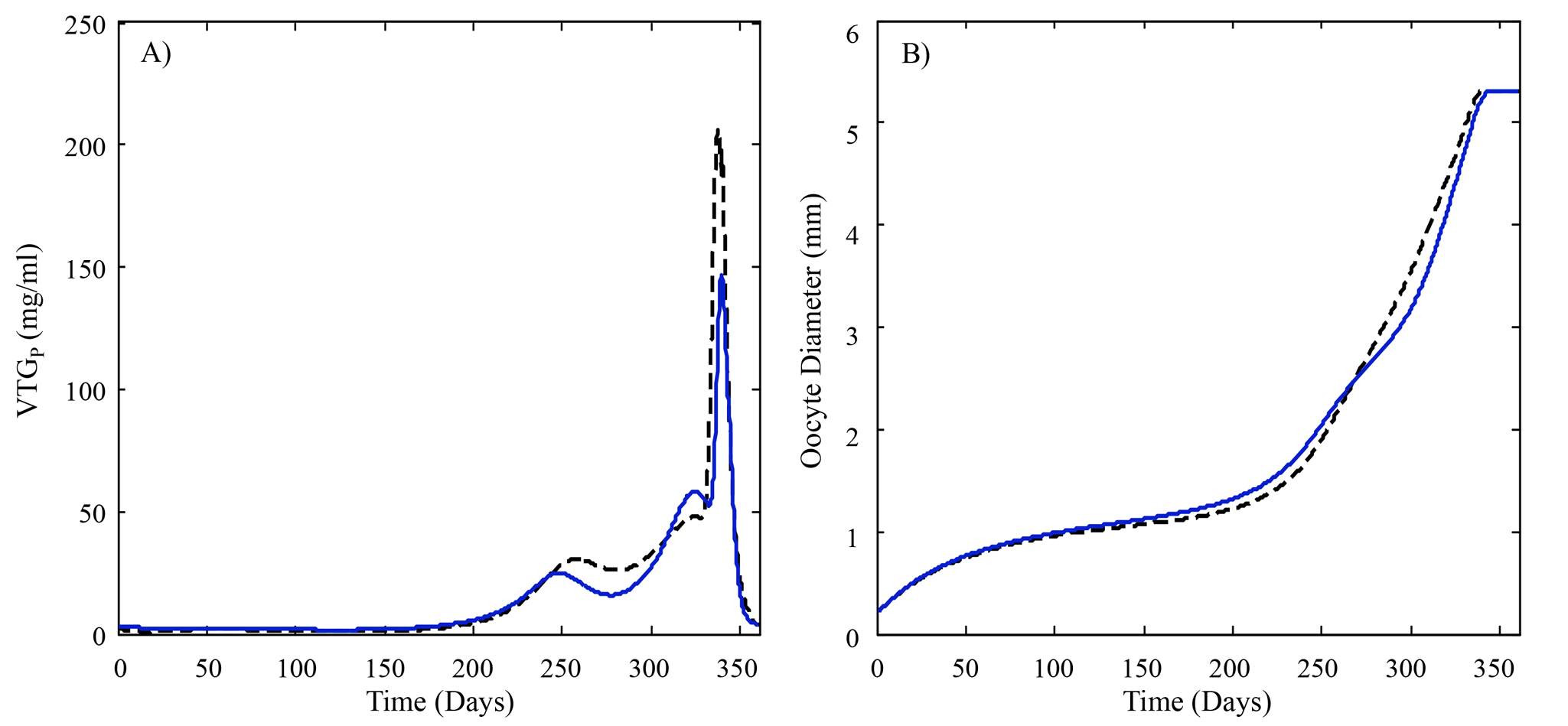

Supplement: S2 Fig — The black dashed line uses the complete VTG model, Eq (21) through Eq (27), to describe E2’s effects on VTG. The blue solid line uses transit compartments to approximate the effects E2 has on VTG using delay of 100 hours. (TIF) [file pcbi.1004874.s002.tif]

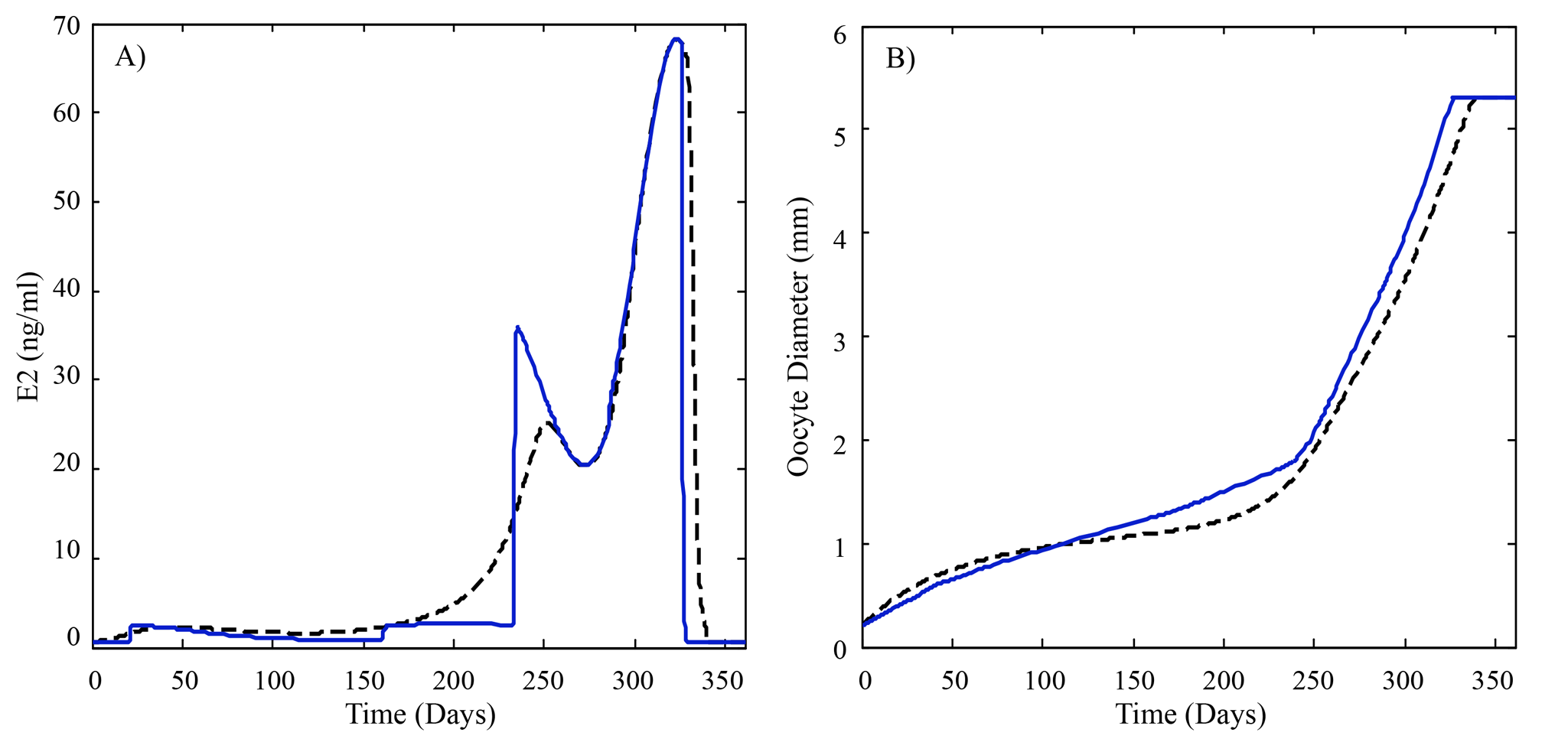

Supplement: S3 Fig — The dashed black line assumes that at any point in time the oocytes could be divided into multiple developmental stages. The blue solid line assumes the developmental stages are segregated. (TIF) [file pcbi.1004874.s003.tif]
