# Supplementary material for: A Computational Model of the Rainbow Trout Hypothalamus-Pituitary-Ovary-Liver Axis
Source: PLoS Comput Biol. 2016 Apr 20;12(4):e1004874. doi: 10.1371/journal.pcbi.1004874 (PMC4838294; doi:10.1371/journal.pcbi.1004874)
Supplement: S1 Appendices — (DOCX) [file pcbi.1004874.s004.docx]

**Supporting Information**

**S1 Appendix: Different Descriptions of Oocyte Growth**

Describing oocyte size by diameter is only one of several approaches used in experimental studies to characterize ovarian growth. The easiest transformation of oocyte diameter would be to model oocyte growth by volume. Assuming a spherical shape, the average volume of the oocytes can be found by using the formula for sphere volume:

$$O_{Vol}=\frac{4}{3}\pi\left(\frac{\left[O_{Avg}\right]}{2}\right)^{3}.$$

Another common way to describe oocyte growth is by using the gonadosomatic index (GSI). To translate the average oocyte diameter to GSI (GSI=Ovary Weight/(Body Weight-Ovary Weight)x100) we used data from [45] and the Curve Fitting Toolbox in MATLAB to obtain

$$GSI=0.3671\left[O_{Avg}\right]^{2.159}+0.1228$$

with error r^2^=0.9475.

**S2 Appendix: Different LH Release Functions**

The rapid non-continuous release of luteinizing hormone (LH) from the pituitary into plasma required a special function, given by

where

$$\Theta(t)=\left[LH_{Pit}\right]-N_{E2}\left[E2\right]-N_{DHP}\left[DHP\right]$$

We also considered alternative equations described below. The model simulations for the different release functions and their effects on circulating LH can be seen in Figure S1 and uses the parameter values listed in Table S1. While R_LH1_(t) is the simplest interpretation of the biological processes involved in the release of LH from the pituitary, it predicts detectible levels of circulating LH over an approximately 40 day period when experimental data shows undetectable levels. A more biologically realistic approach would be to remove restrictions on stage specificity and allow LH release to occur when E2 levels are declining faster than a threshold T_E2_ as shown in Equation :

$$R_{LH2}(t)=\frac{1}{1+e^{-a\cdot\left(-\frac{d}{dt}\left[E2\right]-T_{E2}\right)}}\cdot\left[LH_{Pit}\right].$$

where R_LH2_(t) is a continuous approximation of:

The release functions R_LH2_ has an initial spike, a change from 0 to 30 ng/ml, in circulating levels LH occurring in a few minutes. However, near-instantaneous release of LH into the plasma is not biologically realistic. This led us to consider the release of LH as the spillover of unblocked LH in the pituitary (Equation ). An alternative would be to use a Hill type equation to approximate the Heaviside function, resulting in the release function:

$$R_{LH3}(t)=\left(\frac{\left[LH_{Pit}\right]^{n_{r,LH}}}{\left[LH_{Pit}\right]^{n_{r,LH}}+\left(N_{E2}\left[E2\right]+N_{DHP}\left[DHP\right]\right)^{n_{r,LH}}+1}\right)\left(\frac{\left[E2\right]^{n_{E2,LH}}}{\left[E2\right]^{n_{E2,LH}}+T_{E2,LH}^{n_{E2,LH}}}\right)\cdot\left[LH_{Pit}\right]$$

This function is similar in biological meaning and mathematical output to the release function used in the model; however, predicted levels of pituitary LH that are approximately equal to the amount of dopamine receptors (D2r) needed to maintain the block will result in a positive release regardless of the amount of D2r that is needed to maintain the block. This causes the model to over predict the amount of LH in the plasma. The use of the Heaviside approximation provides similar mathematical results; however, the biological translation of the function differs slightly. Like Equation , LH will be released when the amount of D2r (represented by N_E2_[E2]+N_DHP_[DHP]) is less than the amount needed to maintain the block, i.e. the release will occur when N_E2_[E2]+N_DHP_[DHP]< [LH_P_]. Unlike Equation , LH will be released when the amount of D2r is slightly larger than the amount needed to maintain the block, i.e. the release will also occur when N_E2_[E2]+N_DHP_[DHP]≥[LH_P_]> N_E2_[E2]+N_DHP_[DHP]-ε for some ε>0. This could be rectified by perturbing the amount of D2r needed to maintain the block, but this perturbation would hold no biological meaning and could not be found through experimental means.

| **Table S1: Parameter values for the different LH release functions^1^** | | | |
| --- | --- | --- | --- |
| **Release Function** | **Parameter** | **Value** | **Units** |
| R_LH1_ | k_r,LH_ | 0.001067 | hr^-1^ |
|  | T_E2,LH_ | 1 | ng/ml |
|  | n_E2,LH_ | 6 | unitless |
| R_LH2_ | k_r,LH_ | 0.0023 | hr^-1^ |
|  | T_E2_ | 0.2 | ng/ml |
|  | a | 10^8 | unitless |
| R_LH3_ | k_r,LH_ | 0.007 | hr^-1^ |
|  | n_r,LH_ | 20 | unitless |
|  | N_E2_ | 36.78 | unitless |
|  | N_DHP_ | 2.8 | unitless |
|  | n_E2,LH_ | 9 | unitless |
|  | T_E2,LH_ | 23 | ng/ml |
| ^1^Parameters used E2 data from [45] and DHP and mLH from our experiment (See Figures 2C, 2D and 3B respectively) to create input curves and initial estimates were obtained through optimization methods solving minimum number of differential equation. The final parameter values were refined through optimization methods using experimental data from [45] and our experiment. See the Methods Section for details. | | | |

**S3 Appendix: Using Transit Compartments for E2’s effect on VTG**

Transit compartments are a useful tool to approximate complex biological processes without creating complicated equations. In our model we utilize transit compartments to account for time delays associated with FSH effects on the follicle and with E2’s effects on mLH synthesis. Furthermore, transit compartments can be replaced with submodels, e.g. a steroidogenesis model, to increase the biological detail of the HPOL model.

The current HPOL model uses the detailed submodel given in [12] describing the synthesis of VTG from E2 with a change in the amount of VTG sequestered by the oocytes. With the use of transit compartments we can approximate this biological process and produce similar mathematical results. Figure S2 shows the HPOL model predictions of plasma levels of VTG and average oocyte growth using the detailed VTG model and using the transit compartments to approximate plasma VTG levels. The model predictions use the parameters found in Tables 3 (detailed VTG model) and S2 (the transit compartment approximation). Notice the similarities in the shape of circulating levels of VTG and the ability to predict oocyte growth.

| **Table S2: Parameter values for using transit compartments to approximate E2’s effects on VTG^1^** | | | |
| --- | --- | --- | --- |
| **Parameter** | **Value** | **Units** | **Source** |
| k_E2,VTG_ | 205 | hr^-1^ | Used E2 data from [45] (See Figure 2C)^3,4^ |
| D_E2,VTG_ | 100 | hr | [54]^2,4^ |
| ^1^ Using three transit compartments.  ^2^ Guided initial estimates of the parameter value.  ^3^ Specified data was used to create an input curve and an initial estimate was obtained through optimization methods solving minimum number of differential equation.  ^4^ Final parameter value was refined through optimization methods using experimental data from [45] and our experiment. See the Methods Section for details. | | | |

**S4 Appendix: Non overlapping stages**

The use of overlapping stages provides smoothness to defining stage specific effects in the model. In Figure S3, the stages, S_j_ (j=1,2,…,6,FOM), were defined by the average oocyte size and used the logistic function to approximate the following set of Heaviside functions:

$\begin{matrix} S_{1}(t)=\left\{\begin{matrix} 1 & 0\leq\left[O_{Avg}\right]\leq s_{1} \\ 0 & \text{otherwise} \end{matrix}\right., \\ S_{j}(t)=\left\{\begin{matrix} 1 & s_{j-1}\leq\left[O_{Avg}\right]\leq s_{j} \\ 0 & \text{otherwise} \end{matrix}\right.\text{ for }j=2,\ldots,6\text{, and} \\ S_{FOM}(t)=\left\{\begin{matrix} 1 & s_{6}\leq\left[O_{Avg}\right] \\ 0 & \text{otherwise} \end{matrix}\right. \end{matrix}$, (S6)

Figure S3 shows the model predictions, using the parameter values found in Table 3, for circulating levels of E2 and average oocyte growth. While using segregated staging predicts a similar output to overlapping stages, the plots are not as smooth and contain large jumps.

**S5 Appendix: Model Customization and MATLAB/Octave Code**

The file RainbowTroutModel.m is a program that approximates the solution to the system of ODEs that model the HPOL axis in rainbow trout and is compatible with both MATLAB and Octave (Octave is a free programing language and can be downloaded at https://www.gnu.org/software/octave/). Given the number of successive reproductive cycles the program will return a vector of time points (in hours) with a corresponding solution matrix where each column represents a different protein in the HPOL axis.

Running the code in MATLAB or Octave will require several toolboxes/packages. The toolboxes required to run the code in MATLAB are the Statistics Toolbox and the Curve Fitting Toolbox. The packages required for running the program in Octave can be downloaded at http://octave.sourceforge.net/packages.php and are io, odepkg, splines, and statistics. Both Octave and MATLAB require the files Parameters.xlsx and GnRH.xlsx to run; both of which are included as supplementary files. In the excel file Parameters.xlsx the parameter values listed in Table 3 are in the first column with the corresponding name used in the program in the second column. The excel file GnRH.xlsx has time points (in hours) in the first column with corresponding levels of GnRH in the second column. The GnRH.xlsx file provided in the supplementary data can be used to create a GnRH function that can predict up to three consecutive cycles beginning from the first reproductive cycle.

When fitting the model to the data obtained from [55,62,63], results in Figures 5 and 6, we altered the empirical formula for GnRH and the staging parameters for the vitellogenic stages to create a better fit for the data. The formula for GnRH can be derived from either circulating levels of FSH or mFSH. To create the data points from circulating FSH data points let GnRH = 17.4*FSH and to create the data points from mFSH let GnRH = 2*mFSH. Save the data points with corresponding time points in GnRH.xlsx. The code then uses a shape preserving spline (pchip) to create a function for GnRH.

Altering the staging parameters for the vitellogenic stages is based on the assumption that each stage should account for a certain percentage of the follicle growth. For example, the vitellogenic stage accounts for approximately 84% of the growth in oocyte diameter [5]; therefore,

$\frac{s_{6}-s_{2}}{O_{M}}\approx0.84,$ (S7)

where O_M_ is the maximum average oocyte diameter. An oocyte’s ability to sequester noticeable levels of VTG and enter the vitellogenic stages is dependent on its size, i.e. its diameter must be larger than 0.6 mm [5,41]. Hence, the staging parameter dividing the pre-vitellogenic stages from the vitellogenic stages, s_2_, will remain 0.6 mm. To calculate the proportion of oocyte growth the vitellogenic stages account for we used the maximum average oocyte diameter from [45] and the staging parameter values listed in Table 3. We found that Stages 3, 4, 5 and 6 account for approximately 10%, 9%, 31% and 34% of the growth in oocyte diameter, respectively. The new vitellogenic staging parameters calculated from these percentages still might need minor adjustments.
