# Supplementary material for: A Computational Model of the Rainbow Trout Hypothalamus-Pituitary-Ovary-Liver Axis
Source: PLoS Comput Biol. 2016 Apr 20;12(4):e1004874. doi: 10.1371/journal.pcbi.1004874 (PMC4838294; doi:10.1371/journal.pcbi.1004874)
Supplement: S2 Code — (DOCX) [file pcbi.1004874.s006.docx]

% RainbowTroutModel.m is a program that solves the system of ODEs that

% model the HPOL axis in rainbow trout. This program can be executed with

% either Octave or MATLAB. The output of this program is a vector of

% time points (in hours) with a corresponding solution matrix where each

% column represents a different protein in the HPOL axis. The input is the

% number of successive reproductive cycles you want to predict. The GnRH

% function provided (GnRH.xlsx) will run up to three consecutive cycles

% beginning from the first reproductive cycle.

% Both Octave and MATLAB require the files GnRH.xlsx and

% Parameters.xlsx to run; both of which are included as supplementary

% files. Further explanation of those files and how to customize the model

% for a specific data set can be found in Appendix S5.

% Running the code in MATLAB requires the Statistics Toolbox and the Curve

% Fitting Toolbox. Running the code in Octave requires the following

% packages: io, odepkg, splines, and statistics. When running the code in

% Octave uncomment the line of code which loads the packages needed.

function [Time, Solution]=RainbowTroutModel(cycles)

% Packages needed for Octave. Uncomment the following line when using

% Octave.

% pkg load io; pkg load odepkg; pkg load splines; pkg load statistics;

% Gets the parameters

p=getParameters();

% Creates the function for GnRH

GnRHData=xlsread('GnRH.xlsx'); GnRH=pchip(GnRHData(:,1),GnRHData(:,2));

save GnRHInput.mat GnRH

% End time for one reproductive cycle (approximately 3 weeks after

% ovulation)

endTime = 361; % Days

for i=1:cycles

if i==1

% Initial conditions for the initial spawning cycle

mFSH_0 = 36;

mLH_0 = 0.01;

FSHP_0 = 4.2;

LHPit_0 = 0;

LHP_0 = 0;

E2_0 = 0.5;

DHP_0 = 0;

OAvg_0 = 0.42;

mR_0 = 0;

R_0 = 0;

ER_0 = 0;

mVTG_0 = 0;

VTGL_0 = 0;

VTGP_0 = 0;

VTGN_0 = 0;

DHPGreater_0 = 0;

FOMGreater_0 = 0;

% Note: Transit compartment initial conditions are equal to the

% hormone being delayed.

ICs = [mFSH_0 mLH_0 FSHP_0 LHPit_0 LHP_0 E2_0 DHP_0 OAvg_0 ...

mR_0 R_0 ER_0 mVTG_0 VTGL_0 VTGP_0 VTGN_0 FSHP_0 FSHP_0 ...

FSHP_0 FSHP_0 FSHP_0 FSHP_0 FSHP_0 E2_0 E2_0 E2_0 LHP_0...

DHPGreater_0 FOMGreater_0];

else

% Sets the initial protein levels of the new cycle to the

% protein levels at the end of the previous cycle and resets

% the average oocyte growth for a new batch of oocytes and

% resets the conditions required for ovulation.

ICs = Y(length(Y),:);

ICs(8)=OAvg_0;

ICs(27)=DHPGreater_0;

ICs(27)=FOMGreater_0;

end

% Solves the Differential Equations (time is measured in hours)

BegT=(i-1)*endTime*24; EndT=i*endTime*24;

[T,Y] = ode23s(@(t,y) getDifferentialEquations (t, y, p),...

[BegT EndT],ICs);

if i==1

Time=T;

Solution=Y;

else

Time=[Time;T];

Solution=[Solution;Y];

end

end

end

%% System of Differential Equations

function differentialEquations = getDifferentialEquations(t,y,p)

% Variable names and corresponding position in the solutions matrix

mFSH = y(1); %FSH beta subunit mRNA (Pituitary)

mLH = y(2); %LH beta subunit mRNA (Pituitary)

FSHP = y(3); %Follicle Stimulating Hormone (Plasma)

LHPit = y(4); %Luteinizing Hormone (Pituitary)

LHP = y(5); %Luteinizing Hormone (Plasma)

E2 = y(6); %Estradiol-17beta (Plasma)

DHP = y(7); %17alpha,20beta-hihydroxy-4-pregnen-3-one (Plasma)

OAvg = y(8); %Average oocyte follicle diameter

mR = y(9); %Estrogen receptor mRNA (Liver)

R = y(10); %Estrogen receptor (Liver)

ER = y(11); %Estrogen receptor complex (Liver)

mVTG = y(12); %Vitellogenin mRNA (Liver)

VTGL = y(13); %Vitellogenin protein (Liver)

VTGP = y(14); %Vitellogenin protein (Plasma)

VTGN = y(15); %Vitellogenin protein (Other)

TCFSHP1 = y(16); %Transit compartment FSH to E2 (1)

TCFSHP2 = y(17); %Transit compartment FSH to E2 (2)

TCFSHP3 = y(18); %Transit compartment FSH to E2 (3)

TCFSHP4 = y(19); %Transit compartment FSH to E2 (4)

TCFSHP5 = y(20); %Transit compartment FSH to E2 (5)

TCFSHP6 = y(21); %Transit compartment FSH to E2 (6)

TCFSHP7 = y(22); %Transit compartment FSH to E2 (7)

TCE21 = y(23); %Transit compartment E2 to mLH (1)

TCE22 = y(24); %Transit compartment E2 to mLH (2)

TCE23 = y(25); %Transit compartment E2 to mLH (3)

TCLHP = y(26); %Transit compartment LH to DHP (1)

DHPGreater = y(27); %Total time DHP has been higher than DHP_final

FOMGreater = y(28); %Total time FOM has been higher than FOM_final

% Input value for GnRH - estimated from the experimental data

GnRH = getGnRHInput(t);

% Conditions for Ovulation. When DHP is greater than DHP_final,

% DHPbigger will be positive and when FOM is greater than FOM_final

% then FOMbigger will be positive. When both DHPGreater and FOMGreater

% are positive Ovulation will equal 1, otherwise it is 0. The

% approximation of the Heaviside is used to satisfy uniqueness and

% existence of solutions.

Ovulation=Hsidecont(DHPGreater*FOMGreater);

% Oocyte growth stages

[k,l]=WeibullParameters(OAvg,p,Ovulation);

S1=wblcdf(p.s1,k,l);

S2=wblcdf(p.s2,k,l)-wblcdf(p.s1,k,l);

S3=wblcdf(p.s3,k,l)-wblcdf(p.s2,k,l);

S4=wblcdf(p.s4,k,l)-wblcdf(p.s3,k,l);

S5=wblcdf(p.s5,k,l)-wblcdf(p.s4,k,l);

S6=(wblcdf(p.s6,k,l)-wblcdf(p.s5,k,l))*(1-Ovulation);

SFOM=(1-wblcdf(p.s6,k,l))*(1-Ovulation);

% The sytem of ODEs

differentialEquations = [...

p.ks_mFSH*(1+p.alpha_mFSH_GnRH*GnRH)-p.kd_mFSH*mFSH %1: mFSH

p.ks_mLH*(1+p.alpha_mLH_GnRH*GnRH+p.alpha_mLH_E2*TCE23)-...

p.kd_mLH*mLH %2: mLH

(1/p.V_FSH)*(p.w_Pit*p.ks_FSH*mFSH-p.Cl_FSH*FSHP) %3: FSH_P

p.ks_LH*mLH-p.kd_LH*LHPit-p.kr_LH*(LHPit-p.N_E2*E2-p.N_DHP*DHP)*...

Hside(LHPit-p.N_E2*E2-p.N_DHP*DHP)*...

(E2^p.n_E2/(E2^p.n_E2+p.T_E2_LH^p.n_E2)) %4: LH_Pit

(1/p.V_LH)*(p.w_Pit*p.kr_LH*(LHPit-p.N_E2*E2-p.N_DHP*DHP)*...

Hside(LHPit-p.N_E2*E2-p.N_DHP*DHP)*(E2^p.n_E2/(E2^p.n_E2+...

p.T_E2_LH^p.n_E2))-p.Cl_LH*LHP) %5: LH_P

(1/p.V_E2*(p.n_oocyte*(p.k_E2+TCFSHP7*(p.Cl_E2_S2*S2+...

p.Cl_E2_S3*S3+p.Cl_E2_S4*S4+p.Cl_E2_S5*S5+p.Cl_E2_S6*S6))-...

p.Cl_E2*E2)) %6: E2

(1/p.V_DHP)*(p.n_oocyte*(TCLHP*(p.Cl_DHP_S2*S2+p.Cl_DHP_S3*S3+...

p.Cl_DHP_S4*S4+p.Cl_DHP_S5*S5+p.Cl_DHP_S6*S6+...

p.Cl_DHP_SFOM*(SFOM)))-p.Cl_DHP*DHP) %7: DHP

(p.k_NV_OAvg*(S1+S2+SFOM)+p.k_V_OAvg*p.Cl_VTG_Seq*(1+FSHP/(FSHP+...

p.T_Seq_FSH))*(S3+S4+S5+S6)*VTGP) %8: O_Avg

p.ks_mR*(1+ER*p.alpha_mR_ER)-p.kd_mR*mR %9: mR

p.ks_R*mR-p.kd_R*R-p.kon_ER*E2*R+p.koff_ER*ER %10: R

p.kon_ER*E2*R-(p.koff_ER+p.kd_ER)*ER %11: ER

p.ks_mVTG*(1+p.alpha_mVTG_ER*ER)-p.kd_mVTG*mVTG %12: mVTG

p.ks_VTG*(mVTG/p.N_mVTG)^(p.gamma)-p.kr_VTG*VTGL %13: VTG_L

(p.kr_VTG*VTGL*p.w_L+p.Cl_VTG_Trans*VTGN-(p.Cl_VTG_Trans+...

p.Cl_VTG_Seq*((1+FSHP/(FSHP+p.T_Seq_FSH))*(S3+S4+S5+S6))+...

p.Cl_VTG)*VTGP )/p.V_VTG_P %14: VTG_P

(p.Cl_VTG_Trans*VTGP-p.Cl_VTG_Trans*VTGN)/p.V_VTG_N %15: VTG_N

(7/p.D_FSH_E2)*(FSHP-TCFSHP1) %16: TCFSHP1

(7/p.D_FSH_E2)*(TCFSHP1-TCFSHP2) %17: TCFSHP2

(7/p.D_FSH_E2)*(TCFSHP2-TCFSHP3) %18: TCFSHP3

(7/p.D_FSH_E2)*(TCFSHP3-TCFSHP4) %19: TCFSHP4

(7/p.D_FSH_E2)*(TCFSHP4-TCFSHP5) %10: TCFSHP5

(7/p.D_FSH_E2)*(TCFSHP5-TCFSHP6) %21: TCFSHP6

(7/p.D_FSH_E2)*(TCFSHP6-TCFSHP7) %22: TCFSHP7

(3/p.D_E2_mLH)*(E2-TCE21) %23: TCE21

(3/p.D_E2_mLH)*(TCE21-TCE22) %24: TCE22

(3/p.D_E2_mLH)*(TCE22-TCE23) %25: TCE23

(1/p.D_LH_DHP)*(LHP-TCLHP) %26: TCLHP

Hsidecont(DHP-p.DHP_final) %27: DHPbigger

Hsidecont(SFOM-p.FOM_final) % 28: FOMbigger

];

% Eliminates the small imaginary part sometimes caclulated as a result

% of approximating the solution for the system of odes.

differentialEquations=real(differentialEquations);

end

%% Heaviside function

function Hside = Hside(t)

if t>0

Hside = 1;

else

Hside = 0;

end

end

%% Continuous approximation of the Heaviside function

% Uses the Logistic function and is adjusted to have an ouput of 0 when

% evaluated at t=0.

function Hsidecont = Hsidecont(t)

Hsidecont = 1/(1+exp(-100000000*(t-10^(-3))));

end

%% Parameters for the Weibull Distribution

% Uses Nelder-Mead simplex method optimization to solve for the Weibull

% distribution parameters. The mean is defined by O_Avg and the variance

% is defined by the stages.

function [k l]=WeibullParameters(mean,p,Ovulation)

options = optimset('MaxFunEvals',5000);

pars = fminsearch(@(a) WeibullParameterConditions([a(1),a(2)],mean,...

p,Ovulation),[mean,1],options);

k=pars(1); l=pars(2);

end

function obj = WeibullParameterConditions(abvector,mean,p,Ovulation)

k = abvector(1); l = abvector(2);

[m v]=wblstat(k,l);

if Ovulation<1

S1=wblcdf(p.s1,k,l);

S2=wblcdf(p.s2,k,l)-wblcdf(p.s1,k,l);

S3=wblcdf(p.s3,k,l)-wblcdf(p.s2,k,l);

S4=wblcdf(p.s4,k,l)-wblcdf(p.s3,k,l);

S5=wblcdf(p.s5,k,l)-wblcdf(p.s4,k,l);

S6=wblcdf(p.s6,k,l)-wblcdf(p.s5,k,l);

SFOM=1-wblcdf(p.s6,k,l);

else

S1=0; S2=0; S3=0; S4=0; S5=0; S6=0; SFOM=0;

end

% Oocyte Variance

var=(p.alpha_OVar_S1*S1+p.alpha_OVar_S2*S2+p.alpha_OVar_S3*S3+...

p.alpha_OVar_S4*S4+p.alpha_OVar_S5*S5+p.alpha_OVar_S6*S6)+...

p.alpha_OVar_SFOM*SFOM+p.alpha_OVar_S1*Ovulation;

obj= norm([mean var]-[m v]);

end

%% Parameters for the model

function p = getParameters()

p = struct;

[ParameterValues,ParameterNames]=xlsread('Parameters.xlsx');

for i=1:length(ParameterNames)

p.(ParameterNames{i}) = ParameterValues(i);

end

end

%% Calls the GnRH function

function GnRH = getGnRHInput(t)

load GnRHInput.mat; GnRH=fnval(t,GnRH);

GnRH=(GnRH+abs(GnRH))/2;

end
